# Supplementary material for: Inducing multiple nicks promotes interhomolog homologous recombination to correct heterozygous mutations in somatic cells
Source: Nat Commun. 2023 Sep 15;14:5607. doi: 10.1038/s41467-023-41048-5 (PMC10504326; doi:10.1038/s41467-023-41048-5)
Supplement: Supplementary file 1 — Supplementary Information [file 41467_2023_41048_MOESM1_ESM.pdf]

Supplementary Figures

Inducing multiple nicks promotes interhomolog homologous recombination to correct heterozygous mutations in somatic cells

Akiko Tomita[1], Hiroyuki Sasanuma[2], Tomoo Owa[1], Yuka Nakazawa[3, 4], Mayuko Shimada[3, 4], Takahiro Fukuoka[3, 5], Tomoo Ogi[3, 4] and Shinichiro Nakada[1, 6]*

[1] Department of Bioregulation and Cellular Response, Graduate School of Medicine, Osaka University, Suita, Osaka 565-0871, Japan

[2] Department of Genome Medicine, Tokyo Metropolitan Institute of Medical Science, Tokyo 156-0057, Japan

[3] Department of Genetics, Research Institute of Environmental Medicine (RIeM), Nagoya University, Nagoya 464-8601, Japan

[4] Department of Human Genetics and Molecular Biology, Nagoya University Graduate School of Medicine, Nagoya 464-8601, Japan

[5] Genomedia Inc., Tokyo 113-0033, Japan

[6] Institute for Advanced Co-Creation Studies, Osaka University, Suita, Osaka 565-0871, Japan

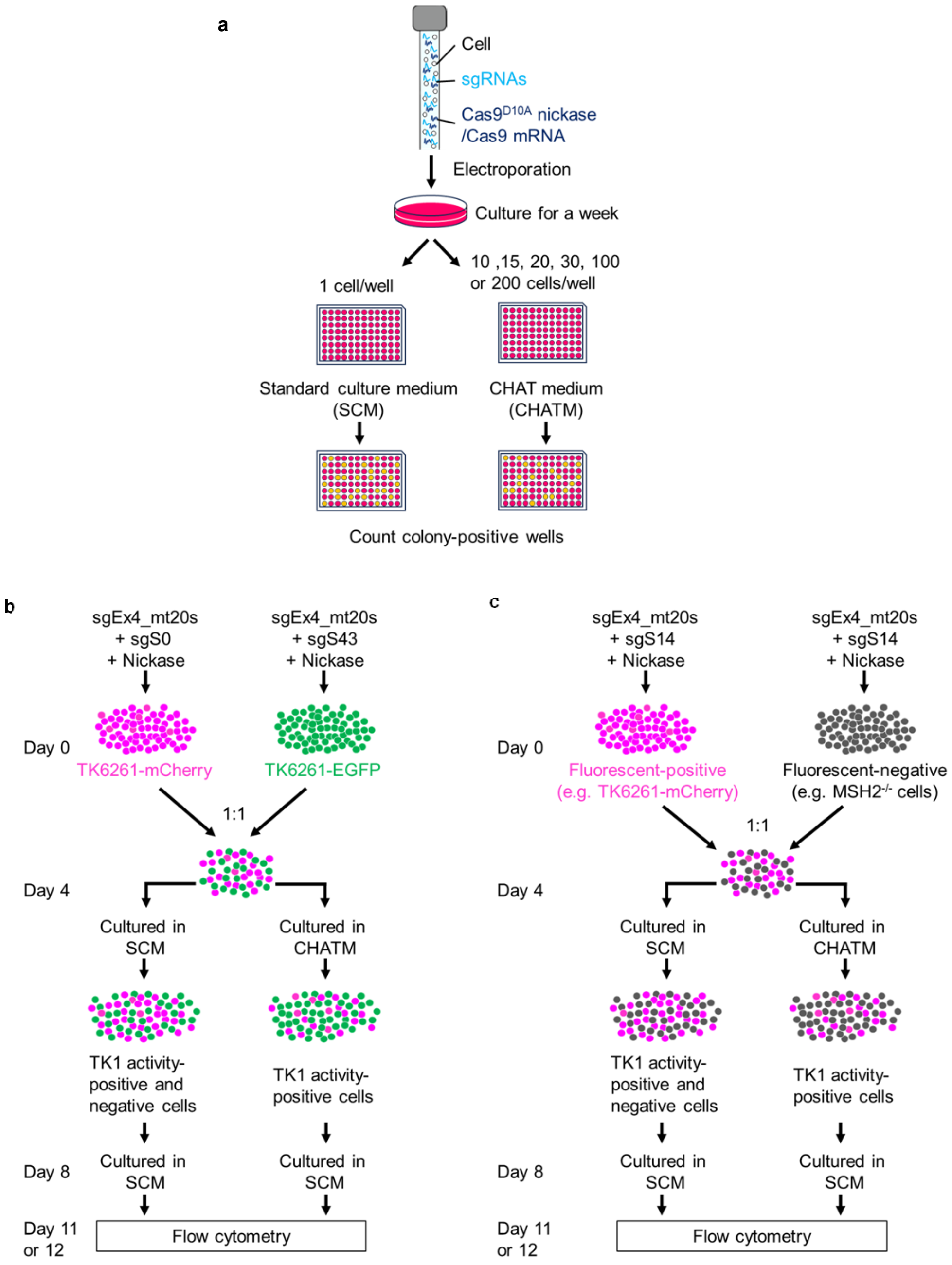

Supplementary Figure 1. Colony formation and proliferation assays. a A schematic illustration of the colony formation assay. Cells were seeded in 200 µL of CHATM at 1, 5, 10, 15, 20, 30, 100, or 200 cells/well in two 96-well plates. To analyze the plating efficiency, cells were seeded in 200 µL of SCM at 1/well in two 96-well plates. Two weeks later, colony-positive wells were analyzed. **b** A schematic illustration of the proliferation assay using TK6261-mCherry and TK6261-EGFP cells. TK6261-mCherry cells were electroporated with sgS0, sgEx4_mt20s, and Nickase. TK6261-EGFP cells were electroporated with Nickase in conjunction with one of the pooled sgRNAs (e.g., S43) and sgEx4_mt20s. After electroporation, TK6261-mCherry and TK6261-EGFP cells were mixed at an approximate 1:1 cell-count ratio. Some of the mixed cells were cultured in SCM, whereas the rest were cultured in CHATM. After culturing in SCM or CHATM for four days and subsequently in SCM for three or four days, mCherry- and EGFP-positive cells were evaluated using flow cytometry. **c** A schematic illustration of the proliferation assay using fluorescence-positive cells (e.g., TK6261-mCherry cells) and fluorescence-negative cells (e.g., $MSH2^{-/-}$ TSCER2 cells). Fluorescence-positive or fluorescence-negative cells were electroporated with sgRNAs and Nickase, as described in each figure and figure legend. After electroporation, fluorescence-positive and fluorescence-negative cells were mixed at an approximate 1:1 cell-count ratio. Some of the mixed cells were cultured in SCM, whereas the rest were cultured in CHATM. After culturing in SCM or CHATM for four days and subsequently in SCM for three or four days, the percentage of fluorescence-positive and fluorescence-negative cells was determined using flow cytometry. The details are provided in the Methods section.

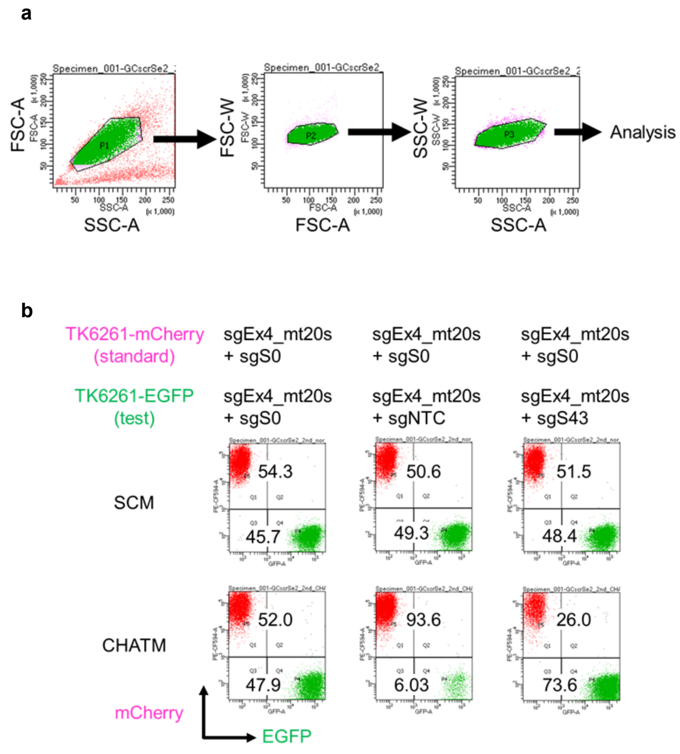

Supplementary Figure 2. Flow cytometry analyses in proliferation assays. a The gating strategy for flow cytometry analyses was as follows: debris, doublets, and atypical cells were excluded through the gates, as shown in the figure. **b** Representative flow cytometry data showing proliferation in CHATM: When both TK6261-mCherry cells and TK6261-EGFP cells were electroporated with sgS0, sgEx4_mt20s, and Nickase, the cell-count ratio of EGFP-positive cells to mCherry-positive cells in the cell populations cultured in SCM was similar to that in CHATM (left panels). When TK6261-EGFP cells were electroporated with sgEx4_mt20s, sgNTC, and Nickase (a combination that lacked the ability to restore TK1 activity; Figure 1e), the ratio of EGFP-positive cells in the cell populations cultured in CHATM was quite low (middle panels). When TK6261-EGFP cells were electroporated with sgS43, sgEx4_mt20s, and Nickase, the TK1 activity was efficiently recovered, and the ratio of EGFP-positive cells was higher than that of mCherry-positive cells in the cell populations cultured in CHATM (right panels).

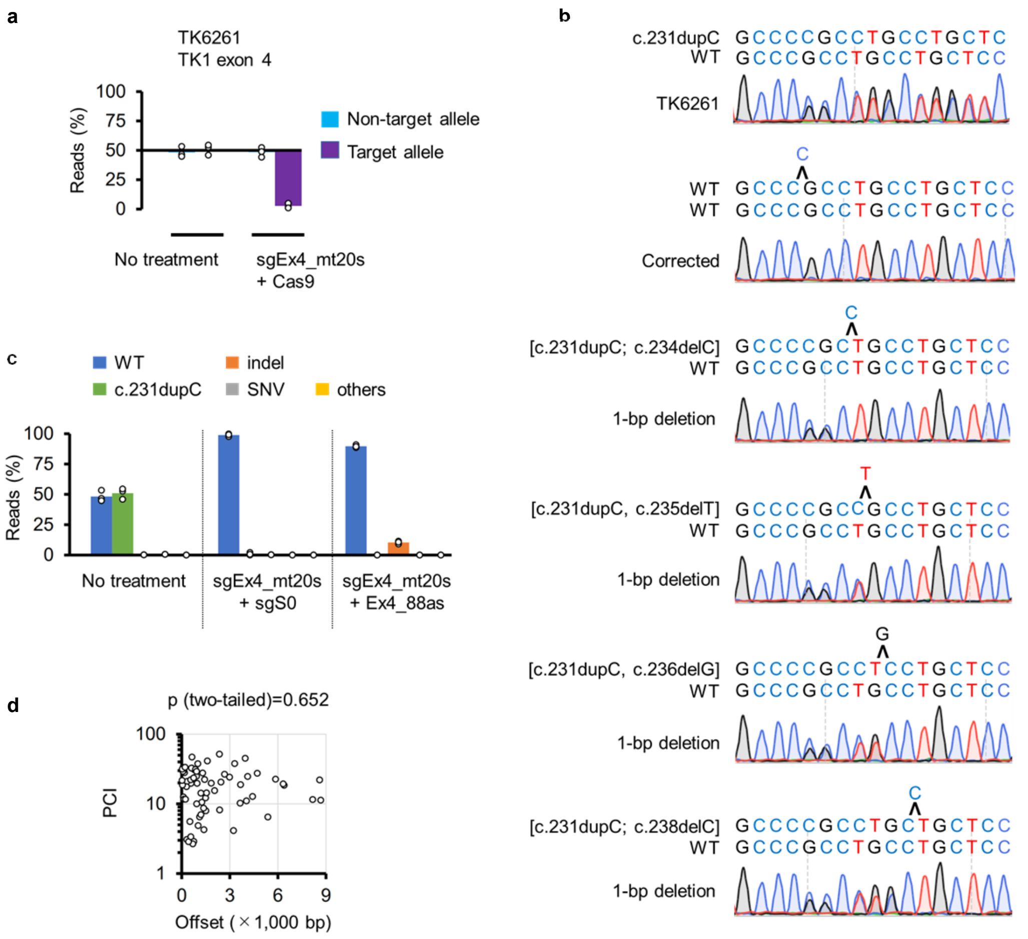

Supplementary Figure 3. Gene editing of the TK1 gene. a AmpNGS of the region surrounding the sgEx4_mt20s target site. Genomic DNA extracted from TK6261 cell populations cultured in SCM after electroporation with sgEx4_mt20s and Cas9 mRNA was analyzed. Percentage of reads that exhibited an identical DNA sequence to either the non-target or target allele (mean ± SD from three independent experiments). The results revealed that only allele A was disrupted, indicating the specific recognition of the mutant sequence by the Cas9-sgEx4_mt20s complex. **b** Representative data from Sanger sequencing. SCCs established in CHATM after gene editing were analyzed. **c** AmpNGS of the region surrounding exon 4 of the TK1 gene (mean ± SD from three independent experiments). Genomic DNA extracted from TK6261 cell populations cultured in CHATM after electroporation with the indicated sgRNAs and Nickase was analyzed. For the no-treatment group, the cells were not subjected to gene editing and were cultured in the SCM. **d** Two-dimensional plot (vertical axis, average PCIs; horizontal axis, offset) of the data shown in Figure 2a. Pearson correlation coefficient was computed. Exact p-value, 0.652; r, -0.05568; 95% confidence interval, -0.2903 to 0.1852. Source data were provided as a Source Data file.

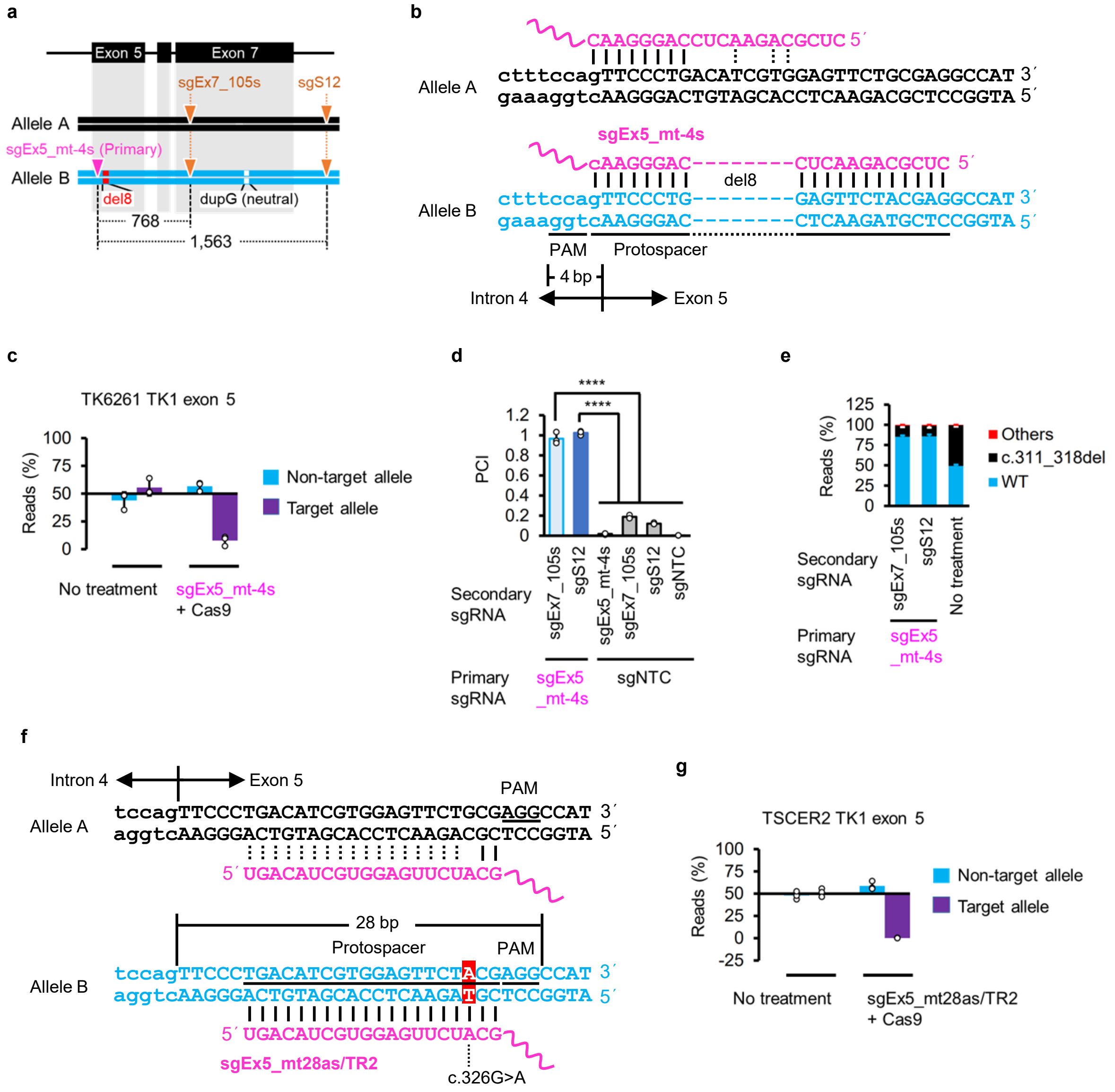

Supplementary Figure 4. Mutations in exon 5 of the TK1 gene were corrected by MNing. a–e Gene correction efficiency determined using a proliferation assay. **a** A schematic illustration of mutations and nicked sites (inverted triangles). del8, c.311_318del; dupG, c.640dupG. **b** A schematic illustration of the genomic DNA sequence and part of the sgEx5_mt-4s sequence. PAM and protospacer sequences are underlined. Possible hydrogen bonds between genomic DNA and sgRNAs are indicated by solid vertical or dashed lines. **c** AmpNGS of the region surrounding the sgEx5_mt-4s target site. Genomic DNA extracted from TK6261 cell populations cultured in SCM after electroporation with sgEx5_mt-4s and Cas9 mRNA was analyzed. Percentage of reads that exhibited an identical DNA sequence to either the non-target or target allele (mean ± SD from three independent experiments). **d** Mean PCI ± SD from three independent experiments. The standard sample was TK6261-mCherry cells electroporated with sgEx5_mt-4s, sgS12, and Nickase. The test samples were TK6261_EGFP cells electroporated with the indicated sgRNAs and Nickase. Data were analyzed using a one-way ANOVA followed by Tukey's multiple comparison test (two-sided). F (5, 12) = 967.2. ****P <0.0001. **e** AmpNGS of the region surrounding the sgEx5_mt-4s target site. Percentage of reads that exhibited an identical DNA sequence to the indicated DNA sequence (mean ± SD from three independent experiments). Genomic DNA extracted from TK6261 cell populations cultured in CHATM after electroporation with the indicated sgRNAs and Nickase was analyzed. **f** A schematic illustration of the genomic DNA sequence of the TK1 gene in TSCER2 cells and part of the sgEx5_mt28as/TR2 sequence. Possible hydrogen bonds between genomic DNA and sgRNAs are indicated by solid vertical or dashed lines. **g** AmpNGS of the region surrounding the sgEx5_mt28as/TR2s target site. Genomic DNA extracted from TSCER2 cell populations cultured in SCM after electroporation with sgEx5_mt28as/TR2s and Cas9 mRNA was analyzed. Percentage of reads that exhibited an identical DNA sequence to either the non-target or target allele (mean ± SD from three independent experiments). Source data were provided as a Source Data file.

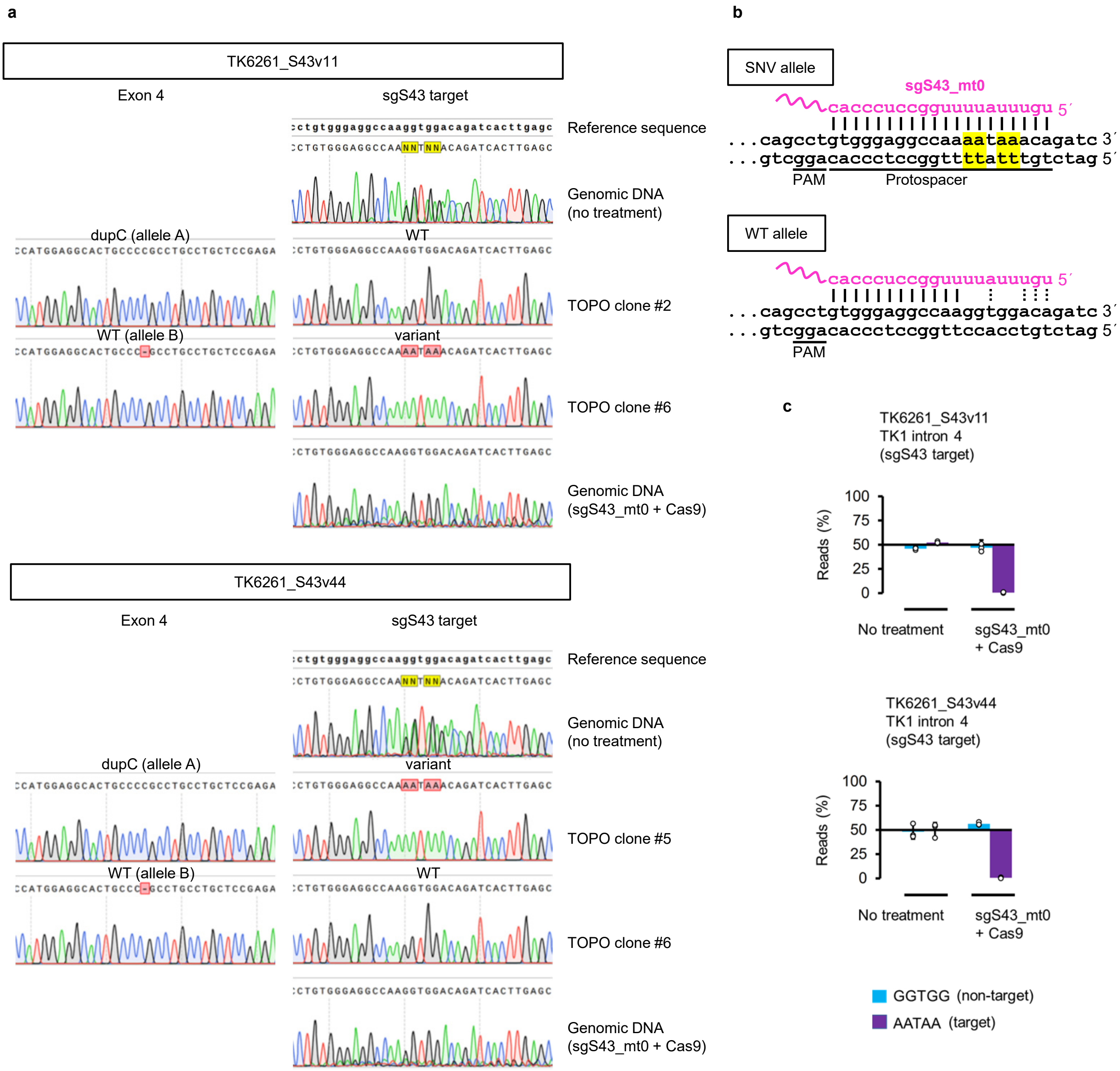

Supplementary Figure 5. TK6261_S43v11 and TK6261_S43v44 cells. a Genotyping of TK6261_S43v11 and TK6261_S43v44 cells. Genomic DNA extracted from the cells was analyzed by Sanger sequencing. The genomic DNA was also subjected to long-range PCR to amplify the region involving exon 4 and the sgS43-target site of the TK1 gene. TOPO clones containing long-range PCR products were analyzed by Sanger sequencing. **b** A schematic illustration of the genomic DNA sequence of the TK1 gene in TK6261_S43v11 or TK6261_S43v44 cells and part of the sgS43_mt0 sequence. Possible hydrogen bonds between genomic DNA and sgRNAs are indicated by solid vertical or dashed lines. **c** AmpNGS of the region surrounding the sgS43_mt0 target site. Genomic DNA extracted from TK6261_S43v11 or TK6261_S43v44 cell populations cultured in SCM after electroporation with sgS43_mt0 and Cas9 mRNA was analyzed. Percentage of reads that exhibited an identical DNA sequence to either the non-target or target allele (mean ± SD from three independent experiments). Source data were provided as a Source Data file.

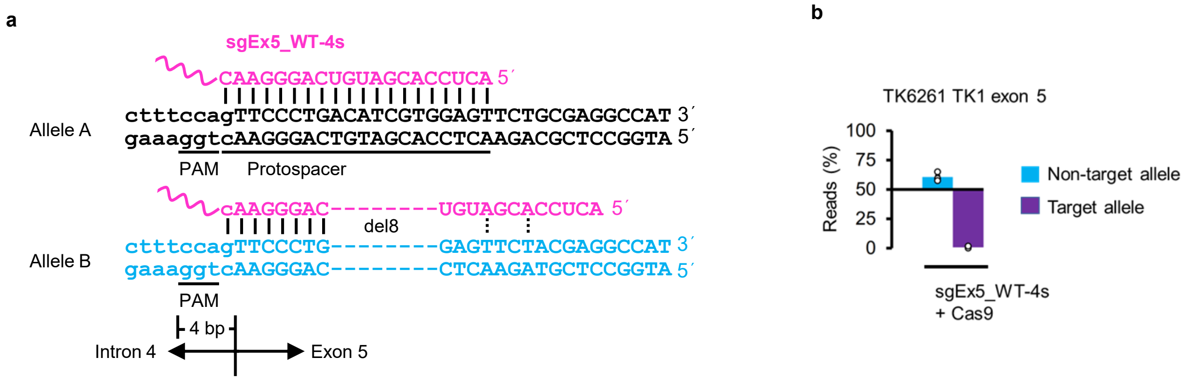

Supplementary Figure 6. Specificity of sgEx5_WT-4s. a A schematic illustration of the genomic DNA sequence of the TK1 gene in TK6261 cells and part of the sgEx5_WT-4s sequence. Possible hydrogen bonds between genomic DNA and sgRNAs are indicated by solid vertical or dashed lines. **b** AmpNGS of the region surrounding the sgEx5_WT-4s target site. Genomic DNA extracted from TK6261 cell populations cultured in SCM after electroporation with sgEx5_WT-4s and Cas9 mRNA was analyzed. Percentage of reads that exhibited an identical DNA sequence to either the non-target or target allele (mean ± SD from three independent experiments). Source data were provided as a Source Data file.

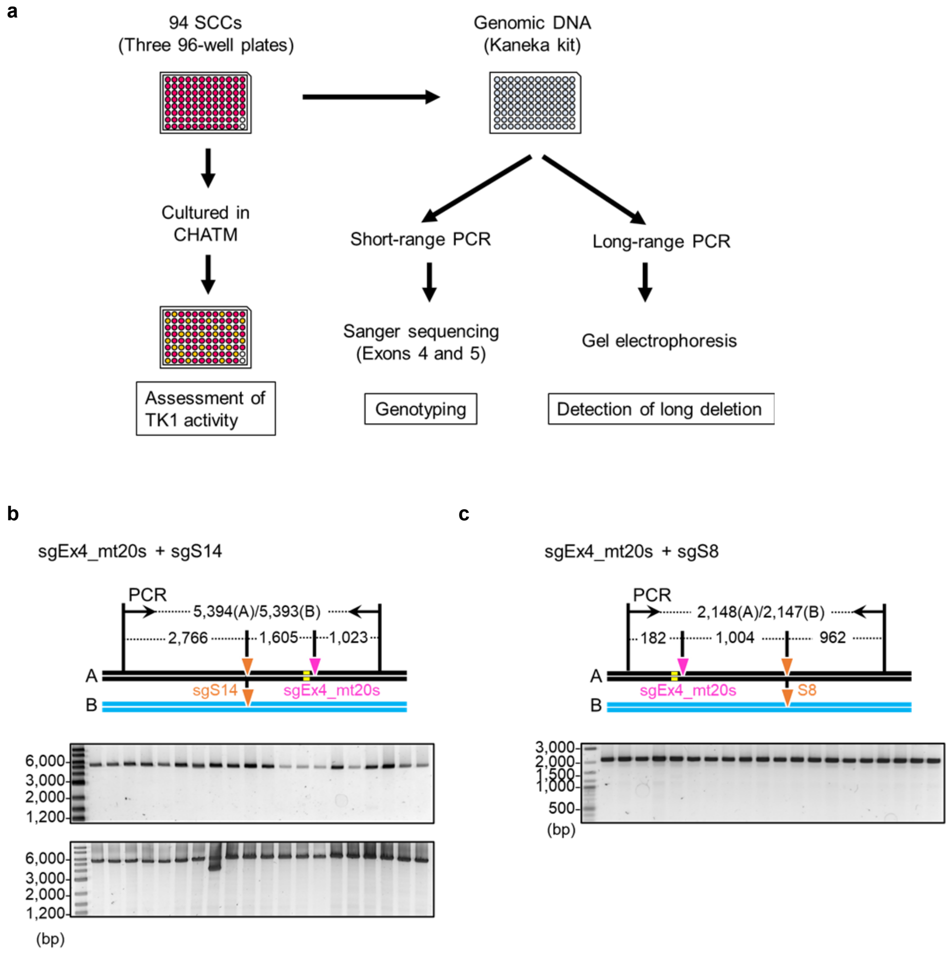

Supplementary Figure 7. Long-range PCR for TK6261 cells. a A schematic illustration of the experimental procedure is shown in Supplementary Figure 8. In total, 282 (94×3) SCCs were established in 3 independent experiments. SCCs were cultured in CHATM to assess the TK1 activity. Genomic DNA was extracted from each SCC using the Kaneka kit and analyzed by Sanger sequencing of exons 4 and 5 of the TK1 gene as well as long-range PCR to amplify regions including the target sites of the sgRNAs. **b, c** Results of long-range PCR. TK6261 cells were electroporated with sgS14, sgEx4_mt20s, and Nickase (**b**) or with sgS8, sgEx4_mt20s, and Nickase (**c**). In the schematic illustration (upper panels), the mutation in exon 4, the target sites of the indicated sgRNAs (inverted triangles), and the primers used for long-range PCR (arrows) are shown. Representative gel electrophoresis results for long-range PCR products (three independent experiments) are shown in the lower panels. Source data were provided as a Source Data file.

a

PCR primer set	Exon 4	Plate number	Total SCC number	WT/dupC	WT/dupC	WT/WT	dupC/dupC	WT	Indel (+)	WT/dupC	others
	Exon 5			WT/del8	WT/del8	WT/del8	WT/del8	WT/del8	WT/del8	del8/del8	
	Long deletion			–	–	–	–	+	–	–	
	TK1 activity			-	+	+	-	-	-	-	
#1	sgEx4_mt20s + sgS14	#2	94	86	0	6	0	2	0	0	0
		#3	94	84	0	7	0	1	1	1	0
		#5	94	85	0	6	0	1	2	0	0
	sgEx4_mt21/WT20s+ sgS14	#1	94	92	0	0	2	0	0	0	0
		#2	94	93	0	1	0	0	0	0	0
		#3	94	93	0	0	0	1	0	0	0
	No treatment	#1	94	94	0	0	0	0	0	0	0
		#2	94	94	0	0	0	0	0	0	0
		#3	94	94	0	0	0	0	0	0	0
#2	sgEx4_mt20s + sgS8	#2	94	89	0	4	0	0	1	0	0
		#4	94	90	0	2	0	1	1	0	0
		#5	94	91	0	2	0	0	1	0	0
	No treatment	#1	94	94	0	0	0	0	0	0	0
		#2	94	94	0	0	0	0	0	0	0
		#3	94	94	0	0	0	0	0	0	0

b

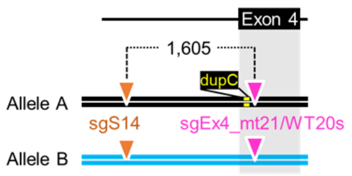

c

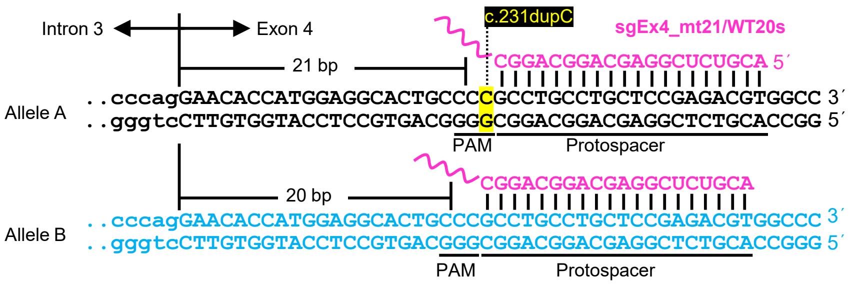

d

Exon 4	Plate number	Total SCC number	WT/dupC	WT/dupC	WT/WT	WT/dupC	WT/WT	Indel (+)	Others
Exon 5			WT/del8	WT/del8	WT/del8	WT/WT	WT/WT	WT/del8	
TK1 activity			–	+	+	+	+	–	
sgEx4_mt20s + sgEx5_mt-4s	#1	94	92	0	1	1	0	0	0
	#2	94	91	0	1	0	2	0	0
	#3	94	91	0	1	0	1	1	0

Supplementary Figure 8. Summary of the results from Sanger sequencing, long-range PCR, and the assessment of the TK1 activity of SCCs. a, d Summary of the results from Sanger sequencing of exons 4 and 5 of the TK1 gene, long-range PCR (**a** only), and an assessment of the TK1 activity in 94 × 3 SCCs. If more than four plates of single cell-derived clones were established, three plates were randomly selected for the analysis. PCR primer set #1: forward 5′-GAGTACTCGGGTTCGTGAACTT, reverse 5′-GCAGCTTCCCATCTATAC-CTCC; PCR primer set #2: forward 5′-GCCTTTCCCATAGGTGCTAACT, reverse 5′-AACAAAACACACTCTGGAAGATGGAACC. dupC, c.231dupC; del8, c.311_318del. **b** A schematic illustration of mutations and nicked sites (inverted triangles). dupC, c.231dupC. **c** A schematic illustration of the genomic DNA sequence of the TK1 gene in TK6261 cells and part of the sgTKex4_mt21/WT20s sequence. Possible hydrogen bonds between genomic DNA and sgRNAs are indicated by solid vertical lines.

a

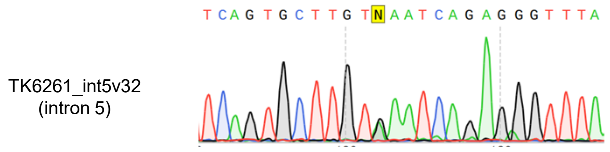

b

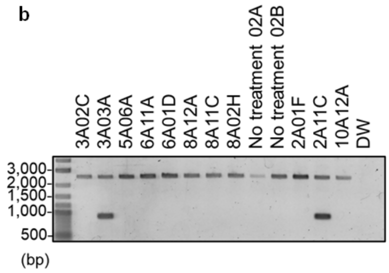

c

Cell clone name	Frequency	Exon 5	Intron 5	Exon 7	Long deletion
2A01F	7/7	WT	G	WT	n.d.
2A11C	6/8	WT	G	WT	n.d.
	2/8	n.s.	n.s.	n.s.	+
3A02C	4/7	WT	G	WT	n.d.
	3/7	WT	A	c.640dupG	n.d.
3A03A	3/7	WT	G	WT	n.d.
	4/7	n.s.	n.s.	n.s.	+
5A06A	8/8	WT	G	WT	n.d.
6A01D	6/6	WT	G	WT	n.d.
6A11A	6/6	WT	G	WT	n.d.
8A02H	6/6	WT	G	WT	n.d.
8A11C	8/8	WT	G	WT	n.d.
8A12A	6/6	WT	G	WT	n.d.
10A12A	2/8	WT	G	WT	n.d.
	6/8	WT	A	c.640dupG	n.d.
no2A	4/8	WT	G	WT	n.d.
	4/8	c.311_318del	A	c.640dupG	n.d.
no2B	1/6	WT	G	WT	n.d.
	5/6	c.311_318del	A	c.640dupG	n.d.

Supplementary Figure 9. Long-range PCR for TK6261 _int5v32 cells. a Exon 5 of the TK1 gene in TK6261_int5v32 cells was analyzed by Sanger sequencing. **b** Gel electrophoresis image from long-range PCR (from two independent experiments). SCCs with WT exon 5 established in SCM after electroporation of TK6261_int5v32 cells with sgEx5_mt-4s, sgS12, and Nickase were analyzed. **c** Genotyping of exon 5, intron 5, and exon 7 of the TK1 gene in the indicated SCCs. Long-range PCR products were inserted into TOPO cloning vectors and analyzed by Sanger sequencing. n.s., no signal; n.d., not detected. Clones no2A and no2B were derived from the untreated TK6261 cells. Source data were provided as a Source Data file.

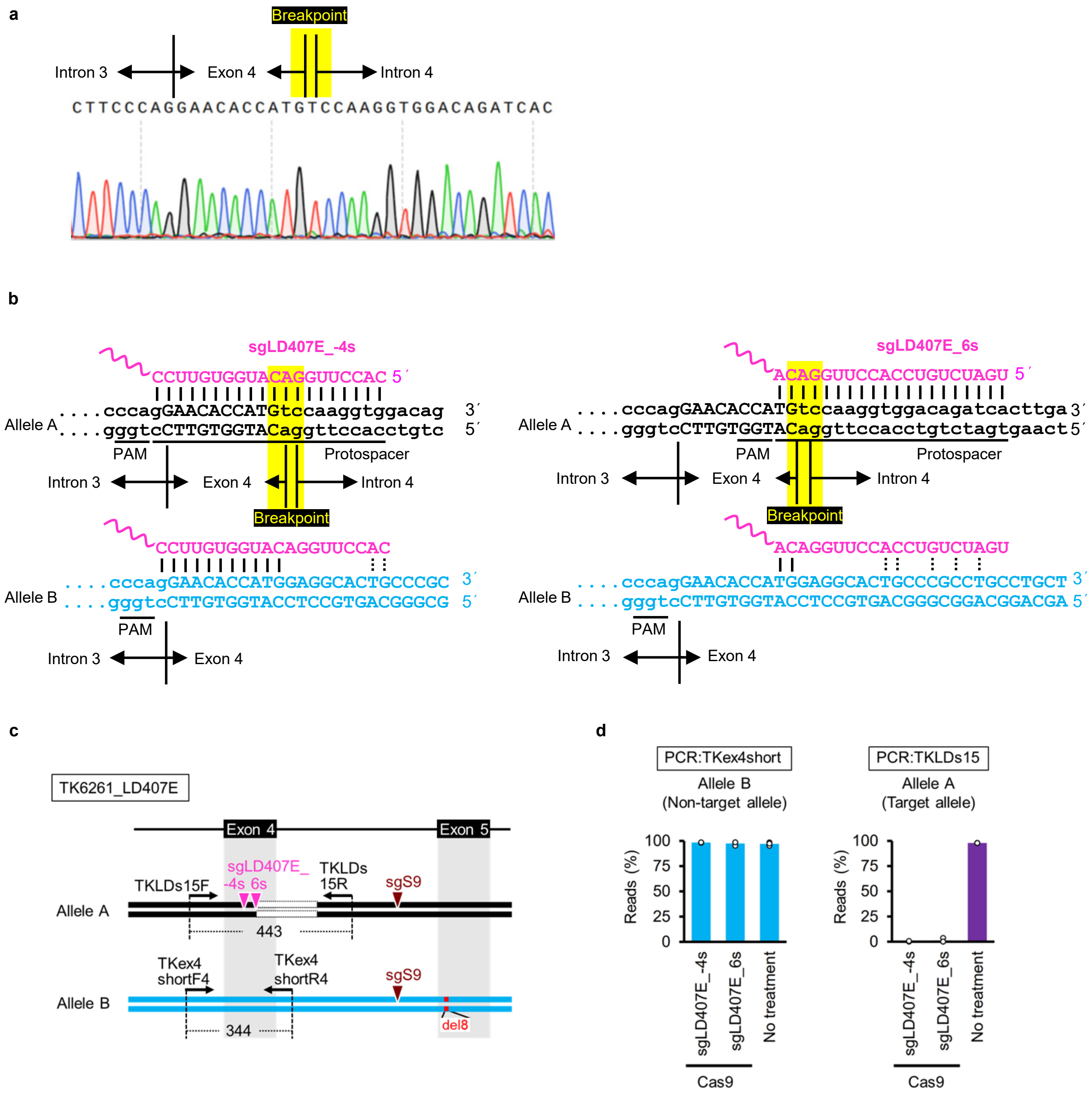

Supplementary Figure 10. TK6261_LD407E cells. **a** Sanger sequencing data showing the breakpoint of the TK1 gene in TK6261_LD407 cells. **b** A schematic illustration of the genomic DNA sequence of the TK1 gene in TK6261_LD407s cells and part of the sgLD407E_-4s or sgLD407E_6s sequence. Possible hydrogen bonds between genomic DNA and sgRNAs are indicated by solid vertical or dashed lines. **c** A schematic illustration of the long deletion of the TK1 gene in TK6261_LD407E cells, target sites of sgRNAs (inverted triangles), and primers for PCR (arrows). del8, c.311_318del. **d** AmpNGS of exon 4 on allele B (left panel) or the region surrounding the target site of sgLD407E_-4s or sgLD407E_6s on allele A (right panel). Genomic DNA extracted from TK6261_LD407E cell populations cultured in SCM after electroporation with sgLD407E_-4s or sgLD407E_6s and Cas9 mRNA was analyzed. The PCR products were electrophoresed on a 1.2% TBE gel, and the shorter PCR products were purified by gel extraction (right panel). Percentage of reads that exhibited an identical DNA sequence to either the non-target (left panel) or target allele (right panel) (mean ± SD from three independent experiments). Source data were provided as a Source Data file.

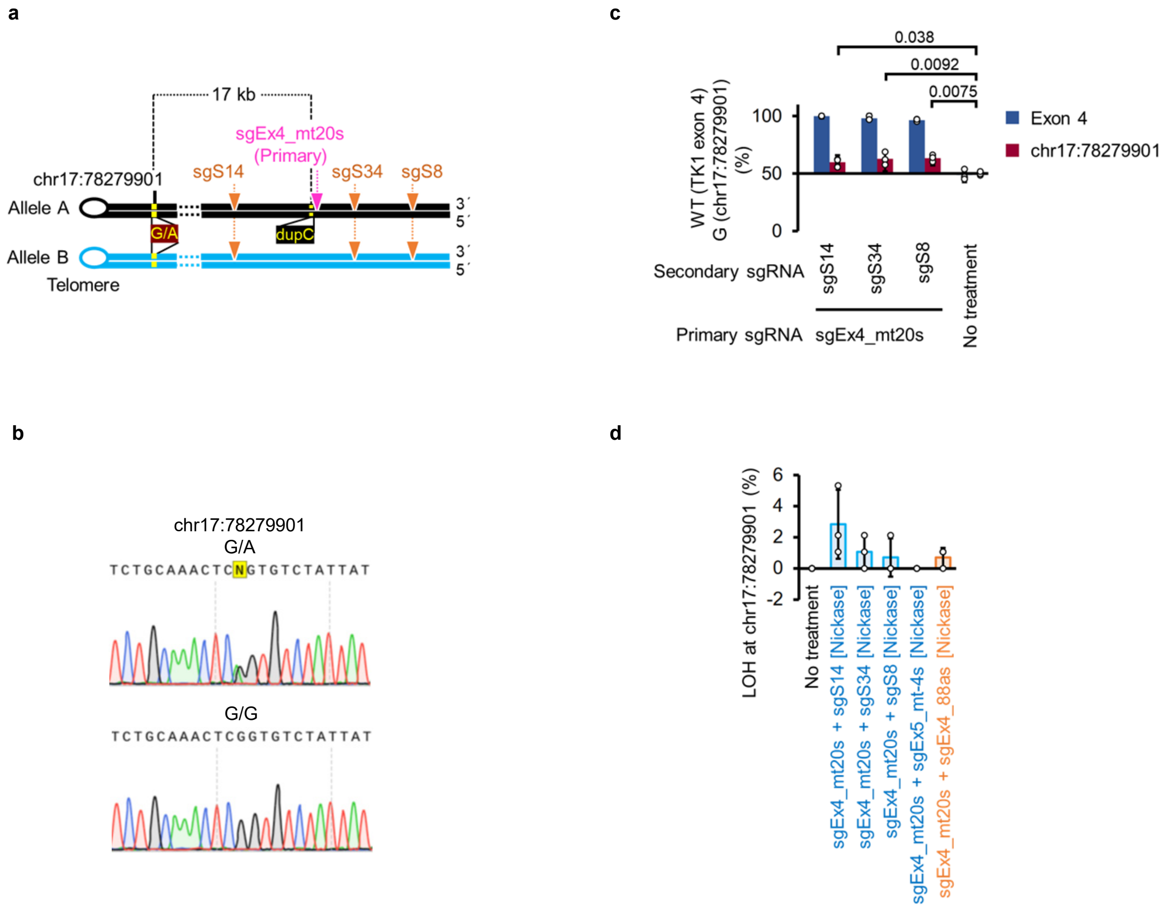

Supplementary Figure 11. Frequency of LOH 17 kb telomeric to the c.231dupC mutation of the TK1 gene. a A schematic illustration of the SNV at Chr17:78279901 (G/A) and the c.231dupC mutation (dupC) in exon 4 of the TK1 gene in TK6261 cells and the target sites of sgRNAs (inverted triangles). **b** Sanger sequencing of the region surrounding Chr17:78279901 in the TK6261 cells. **c** Reads with WT exon 4 sequences or “G” at Chr17:78279901 (%) (mean ± SD from three independent experiments). Data were analyzed using a one-way ANOVA followed by Dunnett's multiple comparison test (two-sided, $F[3, 8]=7.681$). Exact p-values are shown in the graph. **d** Percentage of SCCs with "G" or "A", not "G/A", at Chr17:78279901 in Sanger sequencing. Source data were provided as a Source Data file.

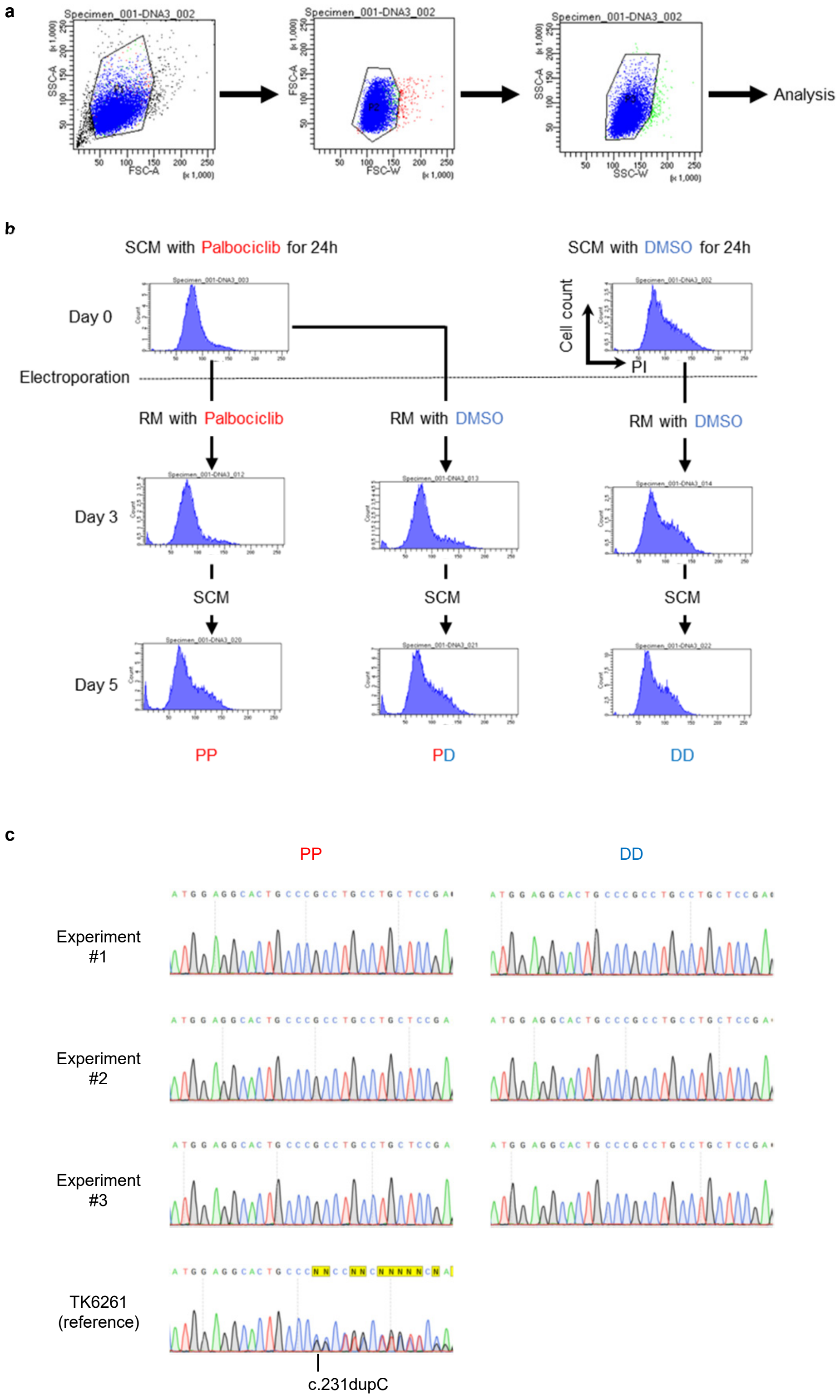

Supplementary Figure 12. A DNA content analysis of cells treated with palbociclib or DMSO by flow cytometry. a The gating strategy for flow cytometry analyses was as follows: debris, doublets, and atypical cells were excluded through the gates, as shown in the figure. **b** Cells were incubated in SCM with palbociclib or DMSO for 24 h prior to electroporation with sgRNA and Nickase. Some cells treated with palbociclib before electroporation were then cultured in recovery medium (RM) containing palbociclib for three days after electroporation. The remaining cells were cultured in RM containing DMSO. Subsequently, cells were cultured in SCM without palbociclib or DMSO. Cells were fixed with paraformaldehyde, treated with RNase A, and stained with propidium iodide for flow cytometry. Data represent three independent experiments. **c** Cells were cultured in CHATM for one week from day 7 and then in SCM for four days. Genomic DNA extracted from the cell populations was analyzed by Sanger sequencing of exon 4 of the TK1 gene.

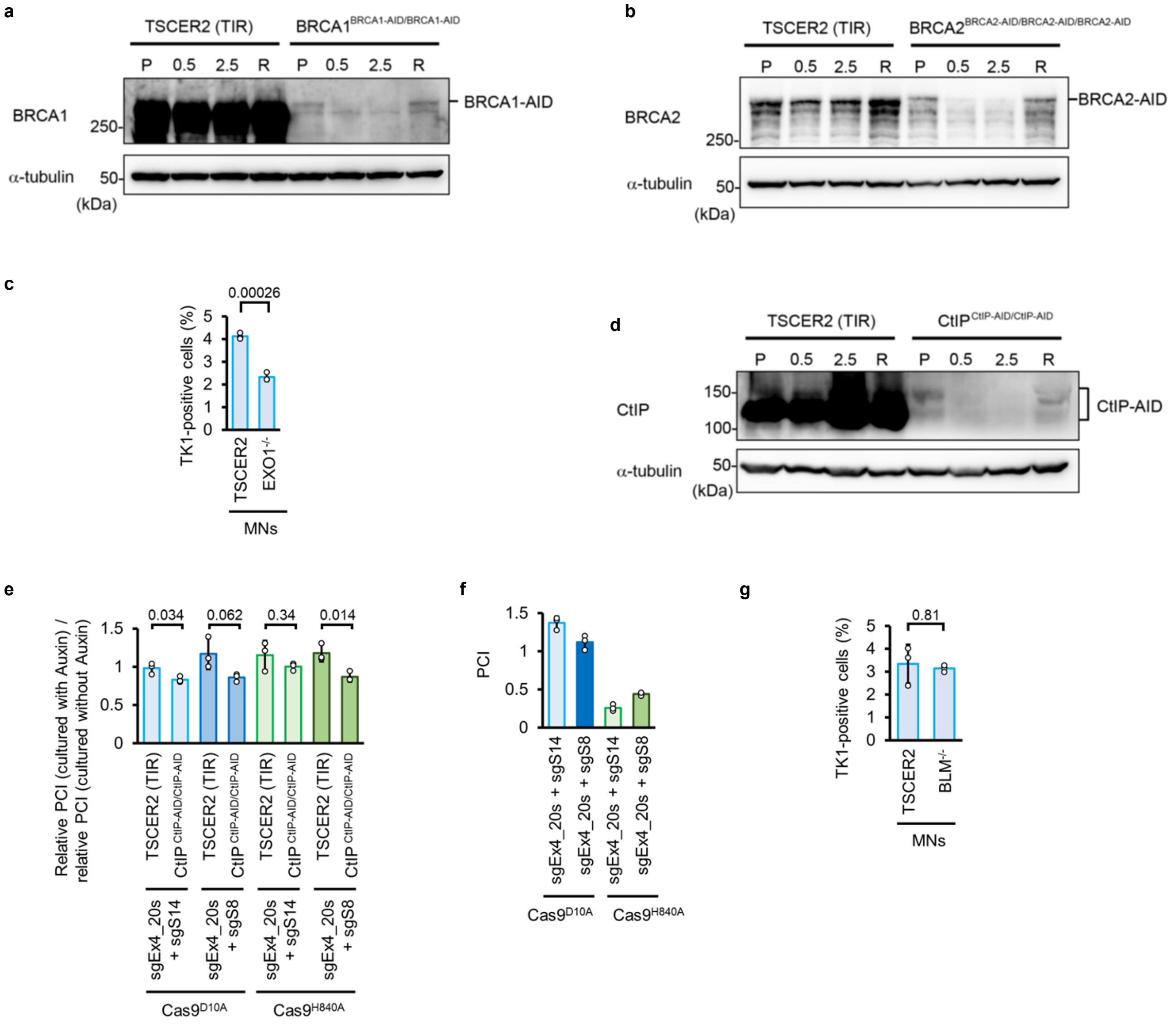

Supplementary Figure 13. Mechanistic insights into MN-IH-HR (1). a, b, d An immunoblot analysis of BRCA1-AID in BRCA1$^{BRCA1\text{-}AID/BRCA1\text{-}AID}$ cells (**a**), BRCA2-AID in BRCA2$^{BRCA2\text{-}AID/BRCA2\text{-}AID/BRCA2\text{-}AID}$ cells (**b**), and CtIP-AID in CtIP$^{CtIP\text{-}AID/CtIP\text{-}AID}$ cells (**d**). Cells were lysed in SDS sample buffer before auxin treatment (P), after half a day of auxin treatment (0.5), after two and a half days of auxin treatment (2.5), and half a day after release from auxin treatment (R). Images are representative of three independent experiments. **c, g** Percentage of TK1 activity-positive cells determined using a colony formation assay. Data represent the mean ± SD from three independent experiments. The data were transformed into a continuous scale using probit transformation and subjected to a two-tailed unpaired *t*-test (**c**: t=12.12, df=4; **g**: t=0.2637, df=4). Exact p-values are shown in the graphs. **e** The ratios of the relative PCI of auxin-treated cells (1 = average PCI in TSCER2 [TIR] treated with auxin) to that of untreated cells (1 = average PCI in TSCER2 [TIR] not treated with auxin) in a proliferation assay. Data represent the mean ± SD from three independent experiments. For the standard samples, TK6261-mCherry cells were used. To deplete CtIP-AID, the cells were treated with auxin beginning half a day prior to electroporation and continuing for two days. The data were transformed into a continuous scale using probit transformation and subjected to a two-tailed unpaired *t*-test (sgEx4_mt20s + S14 + Cas9^{D10A}; t=3.157, df=4, sgEx4_mt20s + S8 + Cas9^{D10A}; t=2.576, df=4, sgEx4_mt20s + S14 + Cas9^{H840A}; t=1.084, df=4, sgEx4_mt20s + S8 + Cas9^{H840A}; t=4.136, df=4). Exact p-values are shown in the graphs. **f** Data represent the mean PCI ± SD from three independent experiments. Cells were electroporated with the indicated sgRNAs and nickase (Cas9^{D10A} or Cas9^{H840A}) mRNA. Source data were provided as a Source Data file.

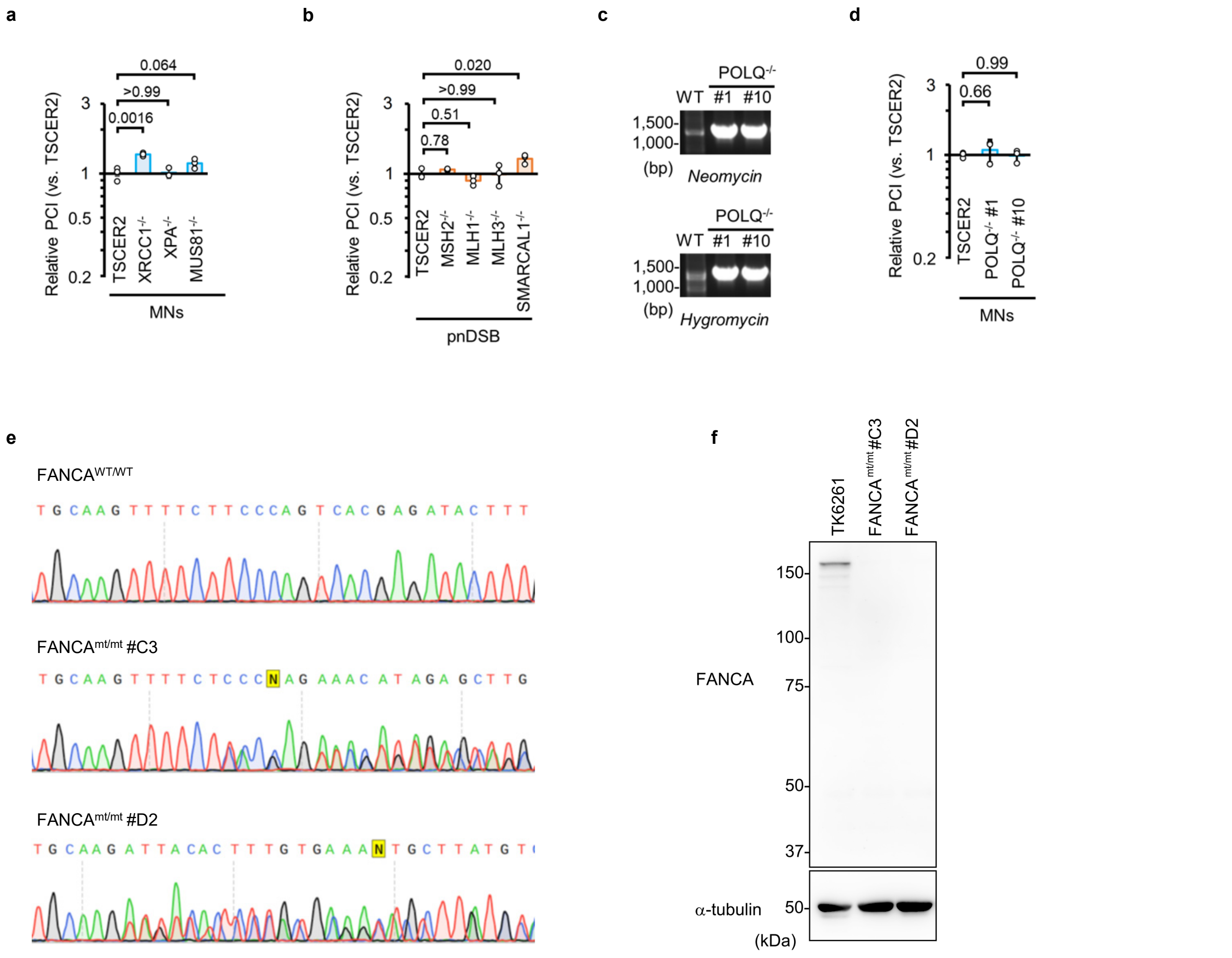

Supplementary Figure 14. Mechanistic insights into MN-IH-HR (2). a, b, d The efficiency of TK1 activity recovery in the indicated gene knockout cells was determined using a proliferation assay. The relative PCIs (1 = average PCI in TSCER2) are shown. The vertical axis represents a logarithmic scale. MNs were introduced using Nickase with sgEx4_mt20s and sgS14 (**a, d**). pnDSBs were introduced using Nickase with sgEx4_mt20s and sgEx4_88as (**b**). The standard sample was TK6261-EGFP cells. Data represent the mean ± SD from three independent experiments. Data were analyzed using a one-way ANOVA followed by Dunnett's multiple comparisons test (two-tailed). F (3, 8) = 13.11 (**a**), F (4, 10) = 6.416 (**b**), and F (2, 6) = 0.4973 (**d**). Exact p-values are shown in the graphs. **c** PCR confirming the successful knock-in of NEOMYCIN^{R} or HYGROMYCIN^{R} at the POLQ loci in POLQ$^{-/-}$ #1 and POLQ$^{-/-}$ #10 cells. Images represent two independent experiments. **e** Sanger sequencing of exon 27 in the FANCA gene. Genomic DNA extracted from FANCA$^{mt/mt}$ #C3 or FANCA$^{mt/mt}$ #D2 cells was analyzed. **f** An immunoblot analysis of FANCA in FANCA$^{mt/mt}$ #C3 and FANCA$^{mt/mt}$ #D2 cells. Images represent three independent experiments. Source data were provided as a Source Data file.

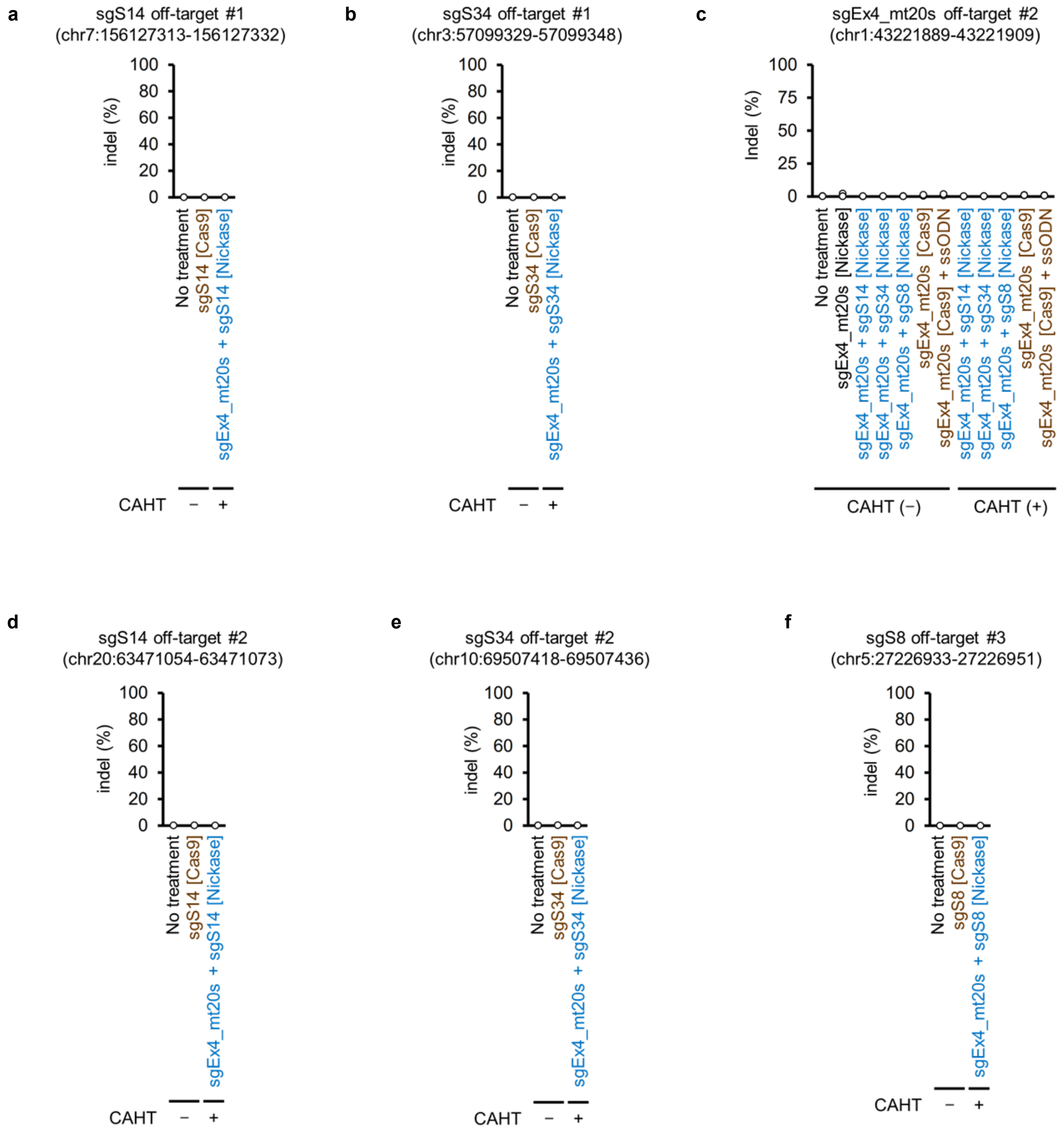

Supplementary Figure 15. Occurrence of short indels at in silico-predicted off-target sites analyzed using AmpNGS. Short indel frequencies in regions surrounding in silico-predicted off-target sites of the indicated sgRNAs. Genomic DNA was extracted from cells electroporated with either Nickase or Cas9 mRNA, accompanied by the respective sgRNAs indicated in each graph, was subjected to ampNGS. CHAT (−), cells cultured in SCM after gene editing; CHAT (+), TK1 activity-positive cells enriched in CHATM after gene editing; ssODN, single-strand oligodeoxynucleotide. Data represent the mean ± SD of three independent experiments. Source data were provided as a Source Data file.

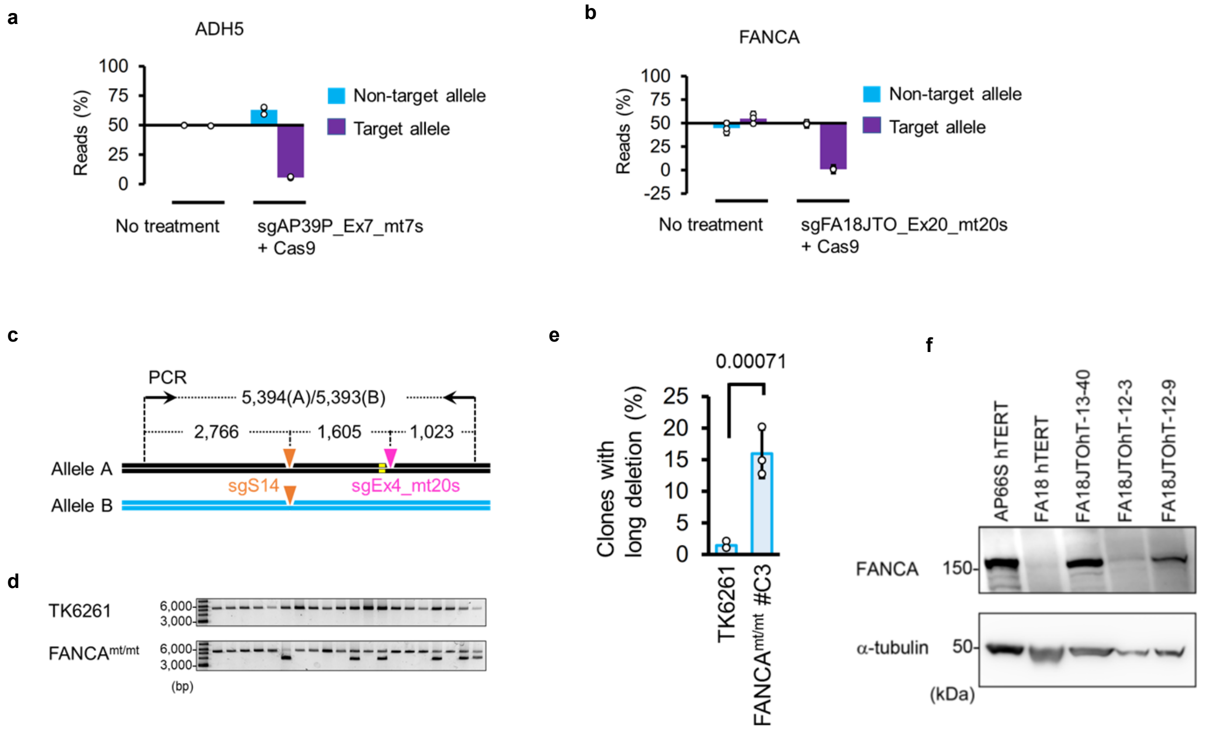

Supplementary Figure 16. Gene correction by NICER in hereditary disease–derived cells. a, b AmpNGS of the region surrounding the sgAP39p_Ex7_mt7s (**a**) or sgFA18JTO_Ex20_mt20s (**b**) target sites. Genomic DNA extracted from AP39P or FA18JTO hTERT cell populations cultured after electroporation with Cas9 mRNA and sgAP39p_Ex7_mt7s (**a**) or sgFA18JTO_Ex20_mt20s (**b**) was analyzed. Percentage of reads that exhibited an identical DNA sequence to either the non-target or target allele (mean ± SD from three independent experiments). **c** A schematic illustration of the exon 4 mutation of the TK1 gene, nicked sites (inverted triangles), and locations of primers for long-range PCR. **d** Representative gel electrophoresis results for long-range PCR products from three independent experiments are shown. **e** Percentage of TK6261 or FANCA$^{mt/mt}$ #C3 cell-derived SCCs with >1,000-bp-long deletions detected using gel electrophoresis of long-range PCR products. Data represent the mean ± SD from three independent experiments. The data were transformed into a continuous scale using probit transformation and subjected to a two-tailed unpaired *t*-test (t=9.422, df=4). Exact p-values are shown in the graph. **f** Immunoblotting for FANCA after NICER (AP66S hTERT cells were used as the FANCA-expressing positive control). Images represent three independent experiments. Source data were provided as a Source Data file.

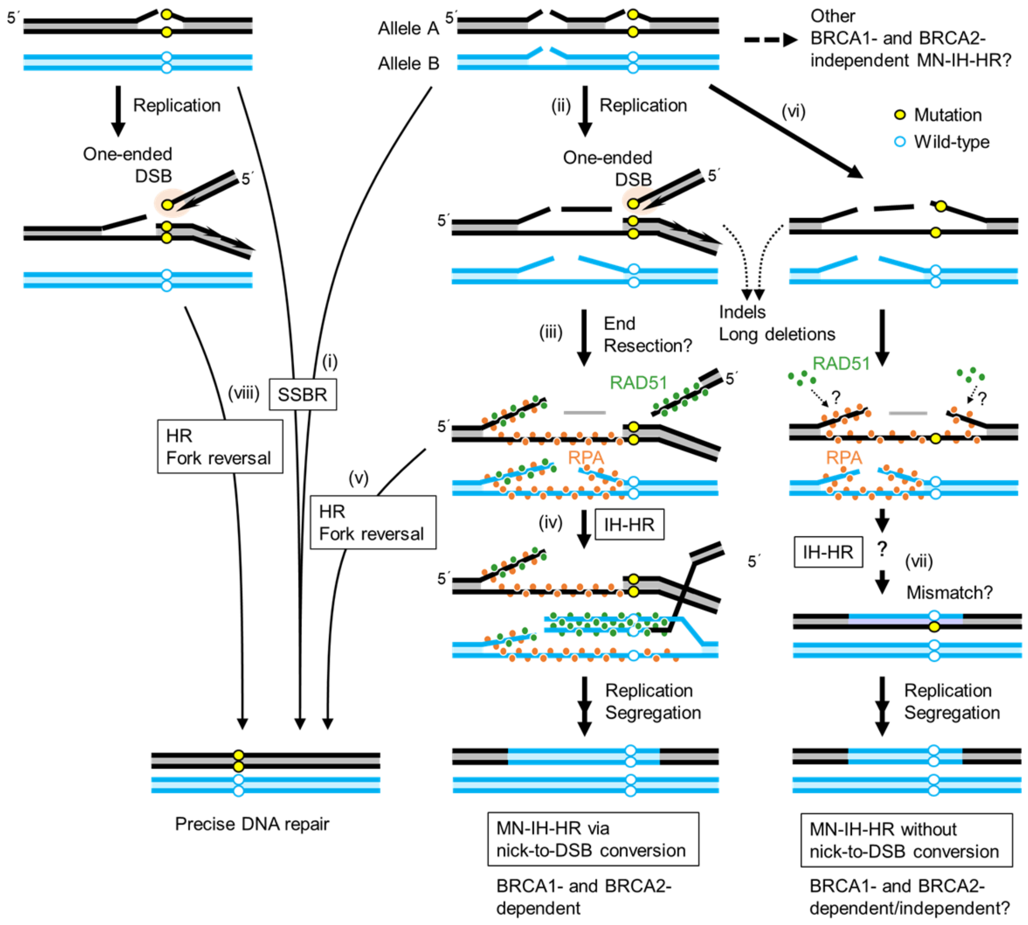

Supplementary Figure 17. Model for a mechanism enabling NICER. Our proposed model for NICER is as follows: (i) Most nicks are repaired via the single-strand break repair (SSBR) pathway. (ii) In the S phase, a replication fork occasionally collides with nickase-induced nicks, converting them into one-ended DSBs. Long single-stranded DNA (ssDNA) strands are exposed on homologous chromosomes. (iii) RPA and RAD51 are loaded onto ssDNA strands. (iv) One-ended DSBs are repaired by BRCA1- and BRCA2-dependent IH-HR, correcting for heterozygous mutations. (v) One-ended DSBs are repaired accurately via HR or fork reversal. (vi) Long ssDNA strands are exposed on both homologous chromosomes. MNs are repaired by MN-IH-HR without nick-to-DSB conversion, correcting for heterozygous mutations. This pathway may be independent of the BRCA1 and BRCA2 expression. However, the detailed molecular mechanisms remain to be elucidated. (vii) In some cases, base mismatches are generated during the MN-IH-HR process. (viii) ssDNA strands are not exposed on a homologous chromosome without a nick (allele B). Therefore, allele B rarely serves as a donor allele, and the nick or one-ended DSB on allele A is repaired by SSBR, HR, or fork reversal. Although numerous variations are possible, the schematic illustration only shows representative models.

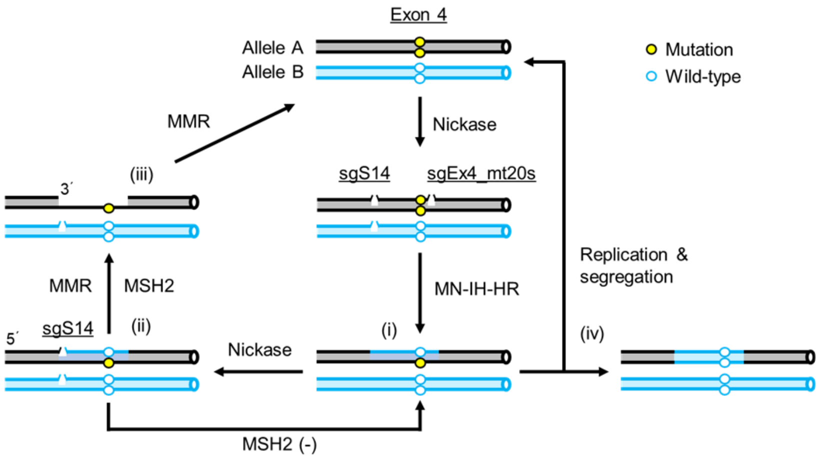

Supplementary Figure 18. MMR-mediated inhibition of MN-IH-HR via correction of a mismatch generated during MN-IH-HR. Our proposed model for MMR-mediated inhibition of MN-IH-HR involves correction of a mismatch generated during MN-IH-HR when TK6261 cells are electroporated with sgEx4_mt20s, sgS14, and Nickase. (i) Only the mutation in exon 4 of the TK1 gene on allele A, which is present on the coding strand, is corrected by incorporating the DNA sequence using allele B as a template. The corrected coding strand reanneals to the non-coding strand of allele A, generating a mismatch. (ii) A nick occurs on the coding strand at the sgS14-targeting site, located 5′ to the mismatch on allele A. (iii) The MMR system misrecognizes the coding strand as the strand with a mutation. The DNA sequence of the coding strand is reverted to the mutant sequence by MMR. Because a nick is generated 5′ to the mismatch, MLH1 and MLH3 are dispensable for MMR in this case. (iv) When a mismatch is not repaired, gene-corrected cells are produced through replication and segregation.

Figure 1g

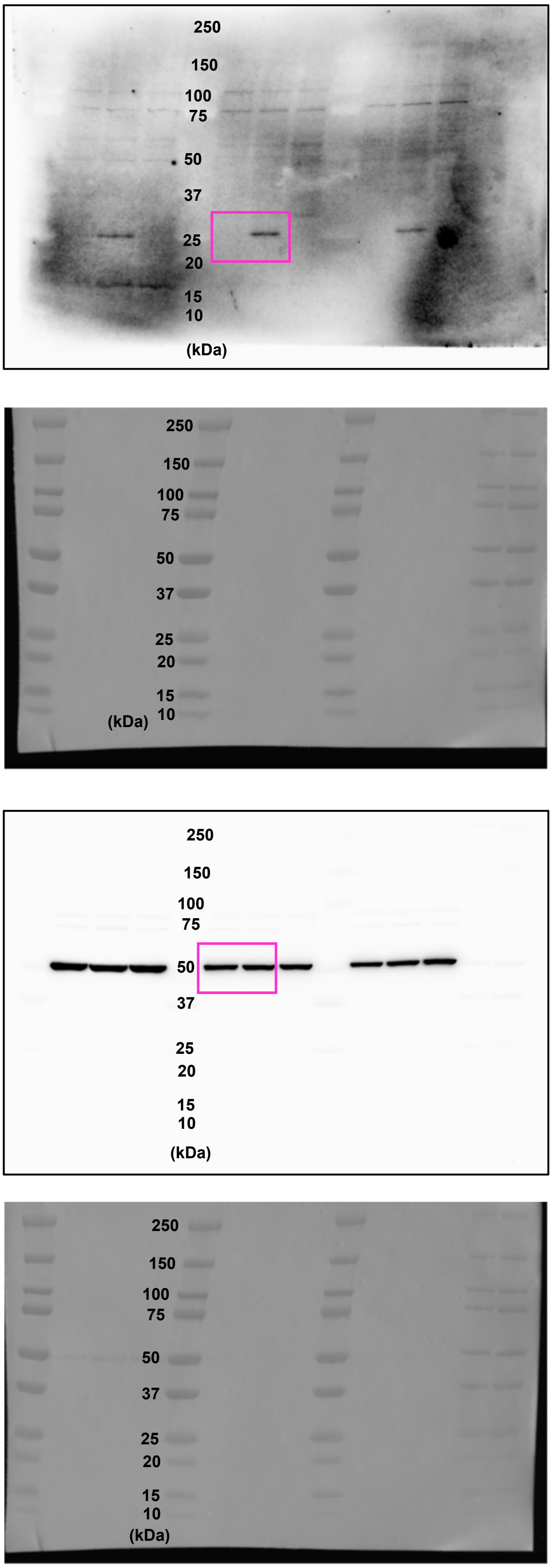

Figure 8d

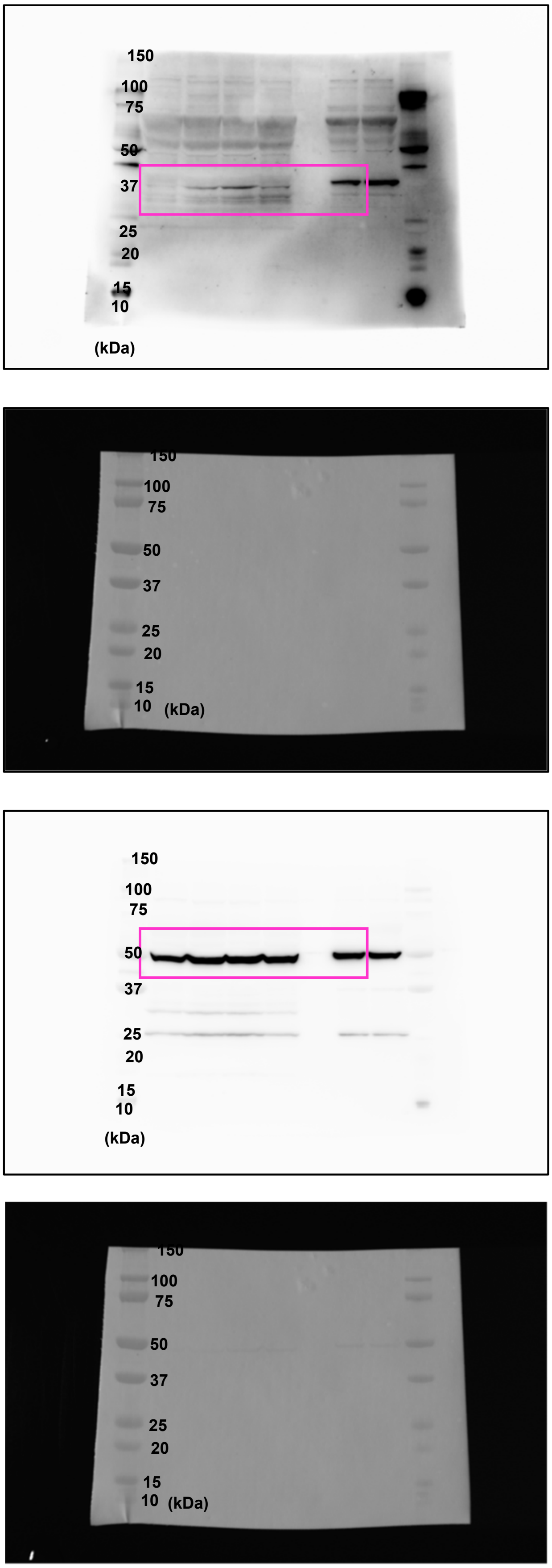

Supplementary Figure 19. Full scan images for Figure 1g and 8d

Figure 8g

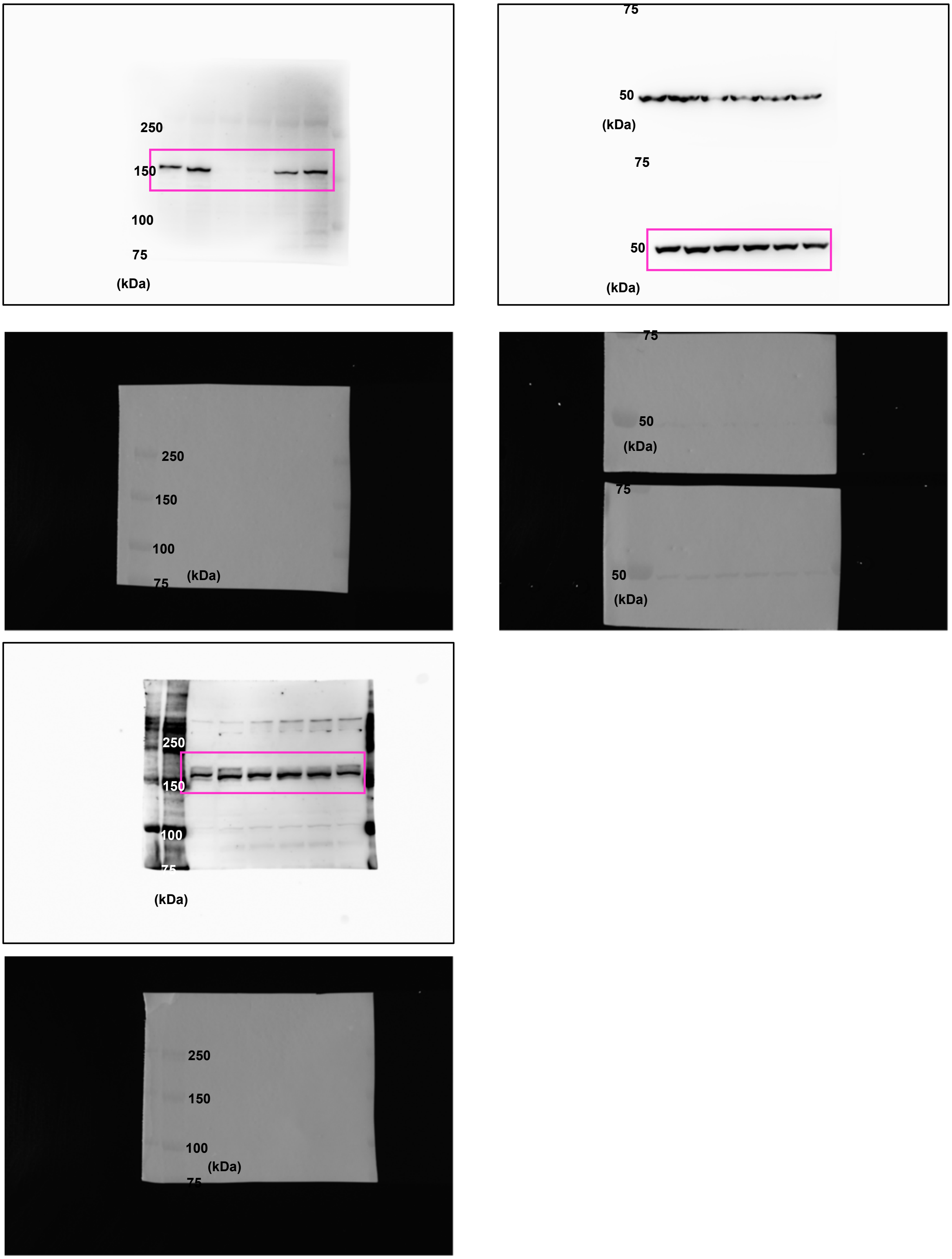

Supplementary Figure 20. Full scan images for Figure 8g

Supplementary Figure 13a

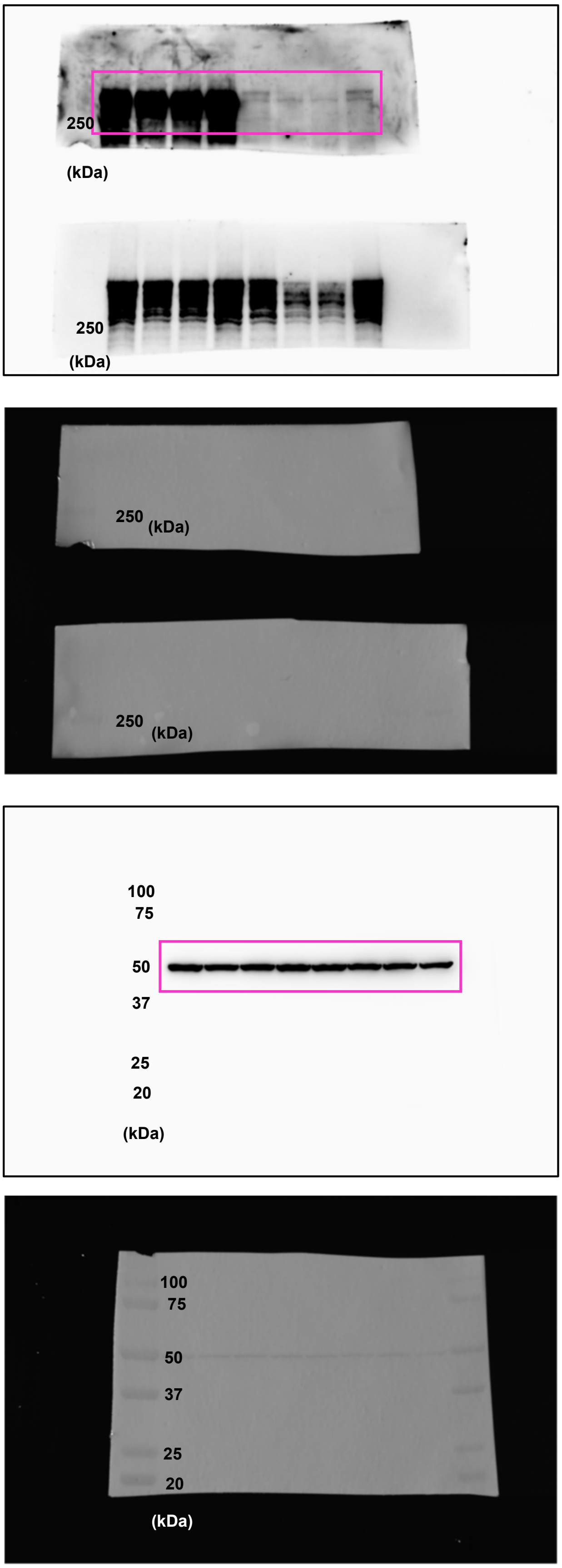

Supplementary Figure 13b

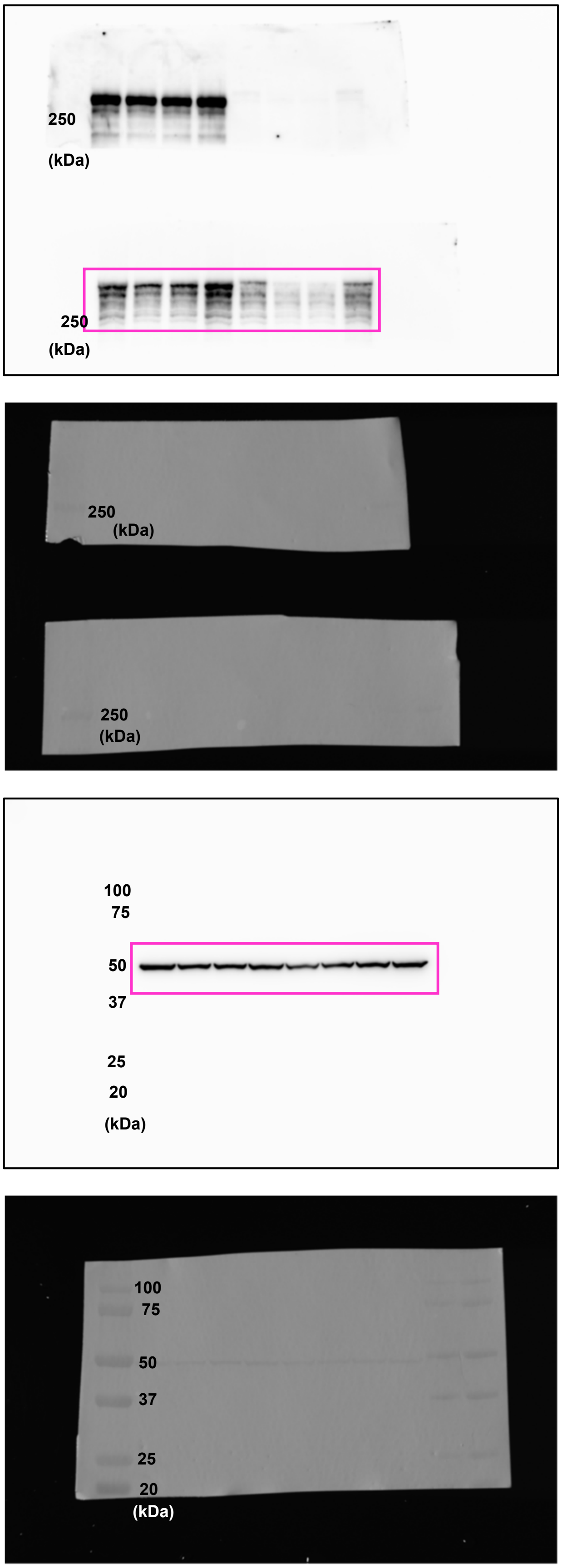

Supplementary Figure 21. Full scan images for Supplementary Figure 13a and b

Supplementary Figure 13d

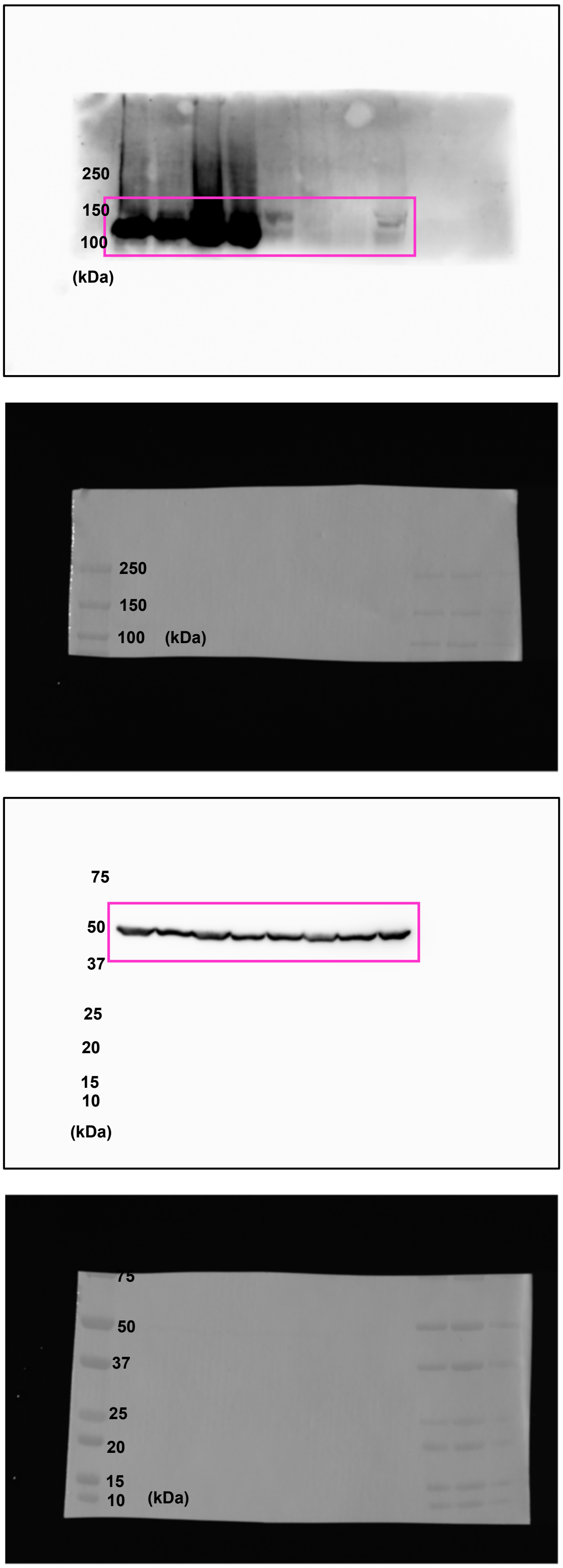

Supplementary Figure 14f

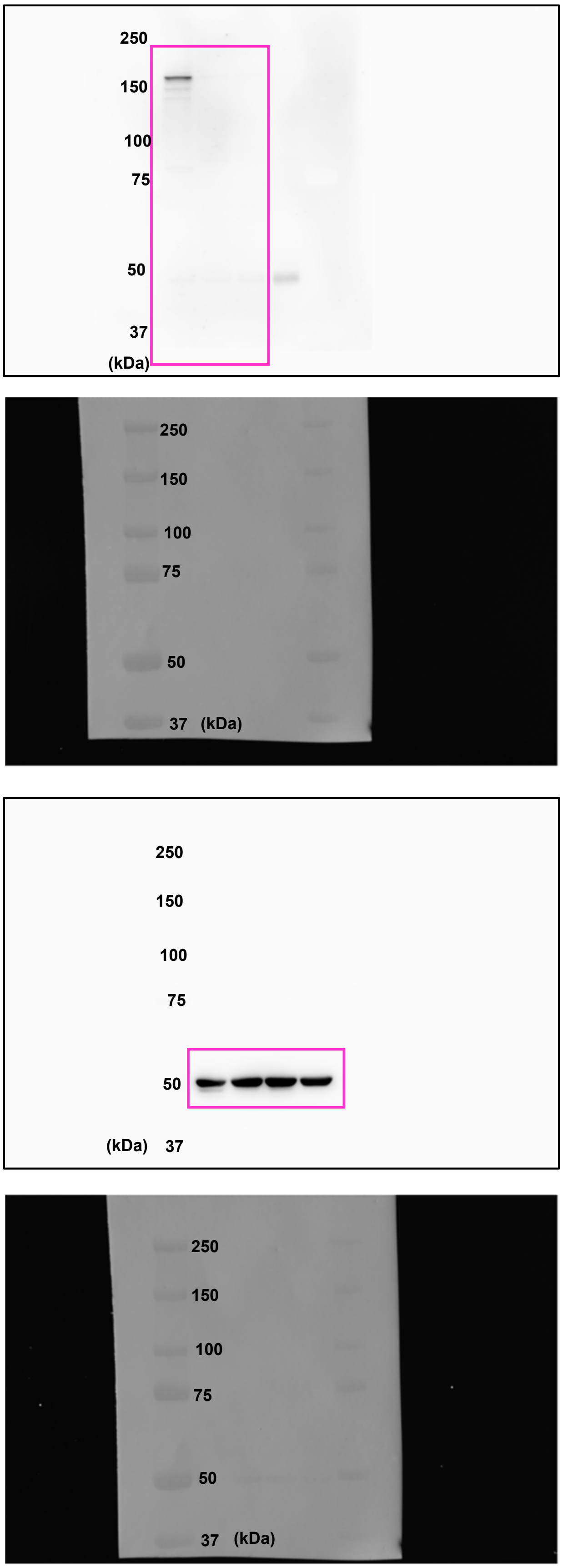

Supplementary Figure 22. Full scan images for Supplementary Figure 13d and 14f

Supplementary Figure 16f

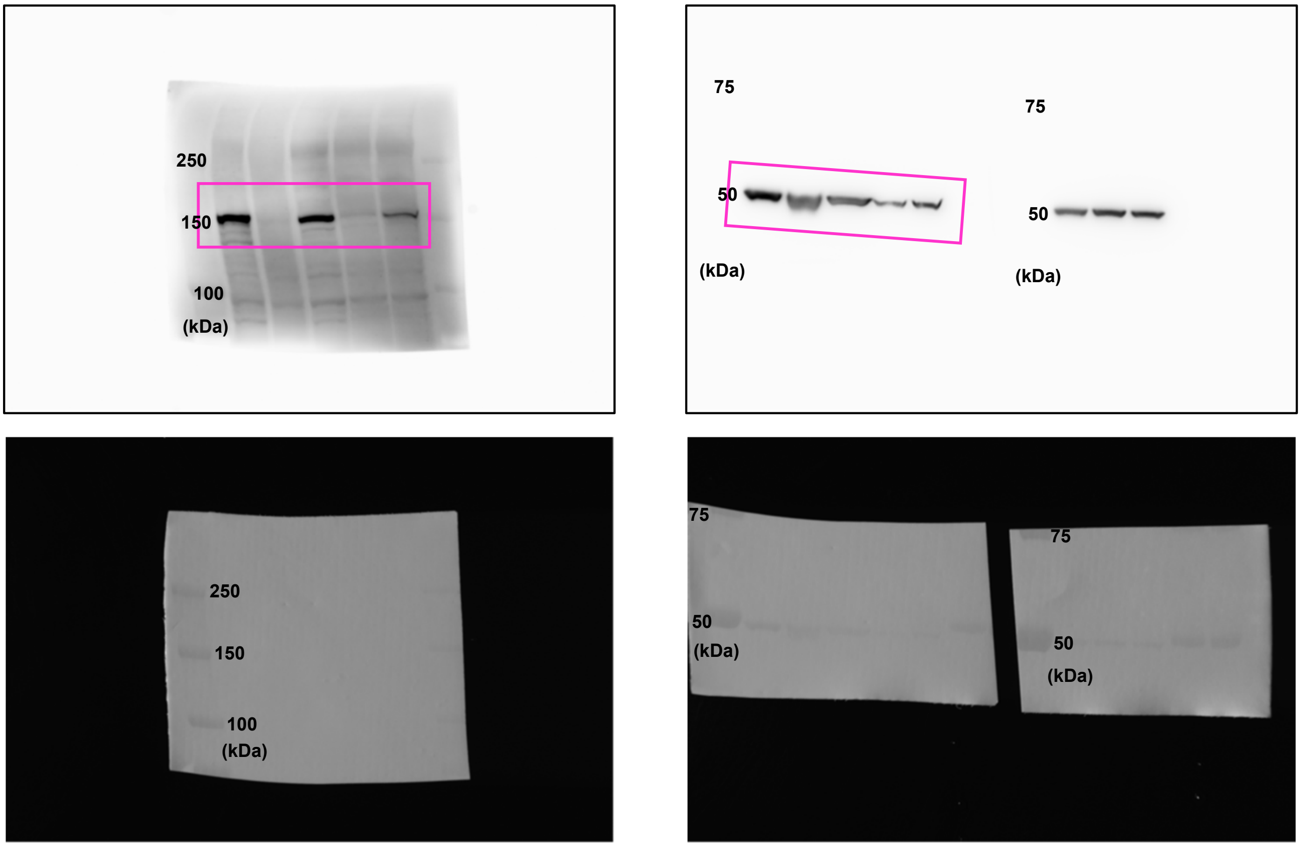

Supplementary Figure 23. Full scan images for Supplementary Figure 16f
