# Supplementary material for: Inducing multiple nicks promotes interhomolog homologous recombination to correct heterozygous mutations in somatic cells
Source: Nat Commun. 2023 Sep 15;14:5607. doi: 10.1038/s41467-023-41048-5 (PMC10504326; doi:10.1038/s41467-023-41048-5)
Supplement: Supplementary file 2 — Reporting Summary [file 41467_2023_41048_MOESM2_ESM.pdf]

nature portfolio

Corresponding author(s): Shinichiro Nakada

Last updated by author(s): Aug 12, 2023

Reporting Summary

Nature Portfolio wishes to improve the reproducibility of the work that we publish. This form provides structure for consistency and transparency in reporting. For further information on Nature Portfolio policies, see our Editorial Policies and the Editorial Policy Checklist.

Statistics

For all statistical analyses, confirm that the following items are present in the figure legend, table legend, main text, or Methods section.

n/a	Confirmed	
☐	☒	The exact sample size (*n*) for each experimental group/condition, given as a discrete number and unit of measurement
☐	☒	A statement on whether measurements were taken from distinct samples or whether the same sample was measured repeatedly
☐	☒	The statistical test(s) used AND whether they are one- or two-sided<br>*Only common tests should be described solely by name; describe more complex techniques in the Methods section.*
☒	☐	A description of all covariates tested
☐	☒	A description of any assumptions or corrections, such as tests of normality and adjustment for multiple comparisons
☐	☒	A full description of the statistical parameters including central tendency (e.g. means) or other basic estimates (e.g. regression coefficient) AND variation (e.g. standard deviation) or associated estimates of uncertainty (e.g. confidence intervals)
☐	☒	For null hypothesis testing, the test statistic (e.g. *F*, *t*, *r*) with confidence intervals, effect sizes, degrees of freedom and *P* value noted<br>*Give P values as exact values whenever suitable.*
☒	☐	For Bayesian analysis, information on the choice of priors and Markov chain Monte Carlo settings
☒	☐	For hierarchical and complex designs, identification of the appropriate level for tests and full reporting of outcomes
☒	☐	Estimates of effect sizes (e.g. Cohen's *d*, Pearson's *r*), indicating how they were calculated

Our web collection on statistics for biologists contains articles on many of the points above.

Software and code

Policy information about availability of computer code

Data collection	FACS Diva (BD, version 8.0.1), ImageQuant LAS4000 (GE, version 1.3), Leica Application Suite Advanced Fluorescence (Leica, version 3.1), QuantaSoft (Bio-Rad, version1.7)
Data analysis	FACS Diva (BD, version 8.0.1), Snapgene (GSL biotech, version 6.2), Genome Analysis Toolkit (McKenna et al., 2010, GATK Queue-3.5), manta program (Illumina, version 1.6.0), BWA (version 0.7.17-r1188), biobambam2 (version 2-2.0.72), Prism (GraphPad, version 9.4), CrispRVariants (Bioconductor, version 1.24.0), R (The R Development Core Team, version 4.3.1), Excel (Microsoft, version 2306), Photoshop (Adobe, version 24.6), sickle (najoshi, version 1.33), Fastx toolkit (Ohio Supercomputer Cente, version 0.0.14), FLASH (JOHNS HOPKINS University Center for Computational Biology, version 2.2.00), fastp program (version 0.20.1),

For manuscripts utilizing custom algorithms or software that are central to the research but not yet described in published literature, software must be made available to editors and reviewers. We strongly encourage code deposition in a community repository (e.g. GitHub). See the Nature Portfolio guidelines for submitting code & software for further information.

Data

Policy information about availability of data

All manuscripts must include a data availability statement. This statement should provide the following information, where applicable:

- Accession codes, unique identifiers, or web links for publicly available datasets
- A description of any restrictions on data availability
- For clinical datasets or third party data, please ensure that the statement adheres to our policy

The AmpNGS data generated in this study have been deposited in the NCBI BioSample database under accession code SAMN36871804- SAMN36872143 [https://www.ncbi.nlm.nih.gov/biosample/36871804-https://www.ncbi.nlm.nih.gov/biosample/36872143]. The NGS data generated in this study have been deposited in the NCBI BioSample database under accession code SAMNXXXXXXXX-SAMNXXXXXXXX [https://www.ncbi.nlm.nih.gov/biosample/XXXXXXX-https://www.ncbi.nlm.nih.gov/biosample/XXXXXXXX]. The other data generated in this study are provided in the Figure/Supplementary Information/Source Data file.

Human research participants

Policy information about studies involving human research participants and Sex and Gender in Research.

Reporting on sex and gender	n/a
Population characteristics	n/a
Recruitment	n/a
Ethics oversight	n/a

Note that full information on the approval of the study protocol must also be provided in the manuscript.

Field-specific reporting

Please select the one below that is the best fit for your research. If you are not sure, read the appropriate sections before making your selection.

[x] Life sciences [ ] Behavioural & social sciences [ ] Ecological, evolutionary & environmental sciences

For a reference copy of the document with all sections, see nature.com/documents/nr-reporting-summary-flat.pdf

Life sciences study design

All studies must disclose on these points even when the disclosure is negative.

Sample size	No statistical method was used to predetermine the sample size. In the colony formation assay, analysis was conducted with 188 wells per experiment per sample, while for Sanger sequencing/long-range PCR/TK1 activity assessment, analysis was performed on 94 single cell-derived clones per sample per experiment. In amplicon-based NGS, a minimum of 30,000 reads (Figure 2b) or 50,000 reads (all other Figures) were analyzed per sample per experiment.
Data exclusions	No data was excluded from analysis.
Replication	All colony formation assays and long-range PCR were repeated in at least three independent experiments. Sanger sequencing, depicted in Figure 1f, was performed once. Sanger sequencing to confirm gene editing efficiency were performed in three independent experiments (as groups). sgRNA pool screening tests using a cell proliferation assay (Figure 2a) were conducted twice. All other proliferation assays were repeated in at least three independent experiments. AmpNGS analyses depicted in Figure 2b were performed once, with 16 similar samples analyzed. Other AmpNGS analyses were repeated in three independent experiments. Immunoblotting experiments were repeated twice of three times.
Randomization	The experiments were not randomized.
Blinding	The investigators were not blinded to the allocation during the experiments and outcome assessment.

Behavioural & social sciences study design

All studies must disclose on these points even when the disclosure is negative.

Study description	*Briefly describe the study type including whether data are quantitative, qualitative, or mixed-methods (e.g. qualitative cross-sectional, quantitative experimental, mixed-methods case study).*
Research sample	*State the research sample (e.g. Harvard university undergraduates, villagers in rural India) and provide relevant demographic information (e.g. age, sex) and indicate whether the sample is representative. Provide a rationale for the study sample chosen. For studies involving existing datasets, please describe the dataset and source.*
Sampling strategy	*Describe the sampling procedure (e.g. random, snowball, stratified, convenience). Describe the statistical methods that were used to predetermine sample size OR if no sample-size calculation was performed, describe how sample sizes were chosen and provide a rationale for why these sample sizes are sufficient. For qualitative data, please indicate whether data saturation was considered, and what criteria were used to decide that no further sampling was needed.*
Data collection	*Provide details about the data collection procedure, including the instruments or devices used to record the data (e.g. pen and paper, computer, eye tracker, video or audio equipment) whether anyone was present besides the participant(s) and the researcher, and whether the researcher was blind to experimental condition and/or the study hypothesis during data collection.*
Timing	*Indicate the start and stop dates of data collection. If there is a gap between collection periods, state the dates for each sample cohort.*
Data exclusions	*If no data were excluded from the analyses, state so OR if data were excluded, provide the exact number of exclusions and the rationale behind them, indicating whether exclusion criteria were pre-established.*
Non-participation	*State how many participants dropped out/declined participation and the reason(s) given OR provide response rate OR state that no participants dropped out/declined participation.*
Randomization	*If participants were not allocated into experimental groups, state so OR describe how participants were allocated to groups, and if allocation was not random, describe how covariates were controlled.*

Ecological, evolutionary & environmental sciences study design

All studies must disclose on these points even when the disclosure is negative.

Study description	*Briefly describe the study. For quantitative data include treatment factors and interactions, design structure (e.g. factorial, nested, hierarchical), nature and number of experimental units and replicates.*
Research sample	*Describe the research sample (e.g. a group of tagged Passer domesticus, all Stenocereus thurberi within Organ Pipe Cactus National Monument), and provide a rationale for the sample choice. When relevant, describe the organism taxa, source, sex, age range and any manipulations. State what population the sample is meant to represent when applicable. For studies involving existing datasets, describe the data and its source.*
Sampling strategy	*Note the sampling procedure. Describe the statistical methods that were used to predetermine sample size OR if no sample-size calculation was performed, describe how sample sizes were chosen and provide a rationale for why these sample sizes are sufficient.*
Data collection	*Describe the data collection procedure, including who recorded the data and how.*
Timing and spatial scale	*Indicate the start and stop dates of data collection, noting the frequency and periodicity of sampling and providing a rationale for these choices. If there is a gap between collection periods, state the dates for each sample cohort. Specify the spatial scale from which the data are taken*
Data exclusions	*If no data were excluded from the analyses, state so OR if data were excluded, describe the exclusions and the rationale behind them, indicating whether exclusion criteria were pre-established.*
Reproducibility	*Describe the measures taken to verify the reproducibility of experimental findings. For each experiment, note whether any attempts to repeat the experiment failed OR state that all attempts to repeat the experiment were successful.*
Randomization	*Describe how samples/organisms/participants were allocated into groups. If allocation was not random, describe how covariates were controlled. If this is not relevant to your study, explain why.*
Blinding	*Describe the extent of blinding used during data acquisition and analysis. If blinding was not possible, describe why OR explain why blinding was not relevant to your study.*

Did the study involve field work? ☐ Yes ☐ No

Field work, collection and transport

Field conditions	*Describe the study conditions for field work, providing relevant parameters (e.g. temperature, rainfall).*
Location	*State the location of the sampling or experiment, providing relevant parameters (e.g. latitude and longitude, elevation, water depth).*
Access & import/export	*Describe the efforts you have made to access habitats and to collect and import/export your samples in a responsible manner and in compliance with local, national and international laws, noting any permits that were obtained (give the name of the issuing authority, the date of issue, and any identifying information).*
Disturbance	*Describe any disturbance caused by the study and how it was minimized.*

Reporting for specific materials, systems and methods

We require information from authors about some types of materials, experimental systems and methods used in many studies. Here, indicate whether each material, system or method listed is relevant to your study. If you are not sure if a list item applies to your research, read the appropriate section before selecting a response.

Materials & experimental systems

n/a	Involved in the study
☐	☒ Antibodies
☐	☒ Eukaryotic cell lines
☒	☐ Palaeontology and archaeology
☒	☐ Animals and other organisms
☒	☐ Clinical data
☒	☐ Dual use research of concern

Methods

n/a	Involved in the study
☒	☐ ChIP-seq
☐	☒ Flow cytometry
☒	☐ MRI-based neuroimaging

Antibodies

Antibodies used

Anti-TK1 (CST, 8960S, 1:1,000 in BSA/TBST for immunoblotting)
Anti-FANCA (CST, 14657S,1:500 in BSA/TBST for immunoblotting)
Anti-FANCD2 (Novus, NB100-182, 1:2,000 in skim milk/TBST for immunoblotting)
Anti-a-tubulin (Sigma-Aldrich, T6074, 1:2,000 in skim milk/TBST for immunoblotting)
Anti-BRCA1 (Santa Cruz, sc-6954 (D-9), 1:100 in skim milk/TBST for immunoblotting)
Anti-BRCA2 (Millipore, OP-95, Ab-1, 1:1,000 in skim milk/TBST for immunoblotting)
Anti-CtIP (Bethyl Laboratories, A300-488A, 1:500 in skim milk/TBST for immunoblotting)
Anti-ADH5 (abcam, ab174283,1:1,000 in skim milk/TBST for immunoblotting)
Anti-ADH5 (Proteintech, 11051-1-AP, 1:500 in skim milk/TBST for immunoblotting)
Anti-RAD51 (Bio Academia, 70-002, lot 1, 10 µL for each ChIP)
Anti-RPA (Merk, MABE285, 3 µg for each ChIP)
Anti-Rabbit IgG (HRP-conjugate) (PROMEGA, W401B, 1:2,000 in skim milk/TBST for immunoblotting)
Anti-Mouse IgG (HRP-conjugate) (PROMEGA, W402B, 1:2,000 in skim milk/TBST for immunoblotting)

Validation

RRIDs in RESEARCH RESOURCE IDENTIFICATION PORTAL (https://scicrunch.org/resources) were indicated
Anti-TK1 (CST, 8960S): AB_2797686
Anti-FANCA (CST, 14657S): AB_2798558
Anti-FANCD2 (Novus, NB100-182): AB_10002867
Anti-a-tubulin (Sigma-Aldrich, T6074): AB_477582
Anti-BRCA1 (Santa Cruz, sc-6954): AB_626761
Anti-BRCA2 (Millipore, OP-95, Ab-1): AB_213443
Anti-CtIP (Bethyl Laboratories, A300-488A): AB_2175262
Anti-ADH5 (Proteintech, 11051-1-AP): AB_593422
Anti-ADH5 (abcam, ab174283) was used in Nakazawa et al. Cell 2020 (PMID: 32142649 DOI: 10.1016/j.cell.2020.02.010).
Anti-RAD51 (Bio Academia, 70-002, lot 1) was used in Nakada et al. Cancer Res 2012 (PMID: 22865450 DOI: 10.1158/0008-5472.CAN-12-1057)
Anti-RPA (Merk, MABE285): AB_11205561
Anti-Rabbit IgG (HRP-conjugate) (Promega, W401B): AB_430833
Anti-Mouse IgG (HRP-conjugate) (Promega, W402B): AB_430834

Eukaryotic cell lines

Policy information about cell lines and Sex and Gender in Research

Cell line source(s)	TK6(IVGT): JCRB JCRB1435<br>TK6261: This paper (S. Nakada, Osaka University, Japan)<br>TK6261_EGFP: This paper (S. Nakada, Osaka University, Japan)<br>TK6261_mCherry: This paper (S. Nakada, Osaka University, Japan)<br>TK6261_S43v11: This paper (S. Nakada, Osaka University, Japan)<br>TK6261_S43v44: This paper (S. Nakada, Osaka University, Japan)<br>TK6261_int5v32: This paper (S. Nakada, Osaka University, Japan)<br>TK6261_S62v49: This paper (S. Nakada, Osaka University, Japan)<br>TK6261_LD407E: This paper (S. Nakada, Osaka University, Japan)<br>TK6261_FANCAmt/mt#C3: This paper (S. Nakada, Osaka University, Japan)<br>TK6261_FANCAmt/mt#D2: This paper (S. Nakada, Osaka University, Japan)<br>TSCER2: Honma et al. Environ Mol Mutagen 2003, PMID: 14673874 (Dr. M. Yasui, National Institute of Health Sciences, Japan)<br>TSCER2: RAD54-/- Hoa et al. Genes Cells 2015, PMID:26525166 (H. Sasanuma, Tokyo Metropolitan Institute of Medical Science, Japan)<br>TSCER2 XRCC1-/-: Saha et al. Environ Mol Mutagen 2018, PMID:29761828 (H. Sasanuma, Tokyo Metropolitan Institute of Medical Science, Japan)<br>TSCER2 XPA-/-: Saha et al. Environ Mol Mutagen 2018, PMID:29761828 (H. Sasanuma, Tokyo Metropolitan Institute of Medical Science, Japan)<br>TSCER2 MUS81-/-: Rahman et al. J Biol Chem 2020, PMID: 3453991 (H. Sasanuma, Tokyo Metropolitan Institute of Medical Science, Japan)<br>TSCER2 BLM-/-: Suzuki et al. Mol Cell Biol. 2016, PMID: 27601585 (Dr. M. Yasui, National Institute of Health Sciences, Japan)<br>TSCER2 MSH2-/-: Rahman et al. J Biol Chem 2020, PMID: 3453991 (H. Sasanuma, Tokyo Metropolitan Institute of Medical Science, Japan)<br>TSCER2 MLH1-/-: Rahman et al. J Biol Chem 2020, PMID: 3453991 (H. Sasanuma, Tokyo Metropolitan Institute of Medical Science, Japan)<br>TSCER2 MLH3-/-: Rahman et al. J Biol Chem 2020, PMID: 3453991 (H. Sasanuma, Tokyo Metropolitan Institute of Medical Science, Japan)<br>TSCER2 SMARCAL1-/-: Keka et al. Nucleic Acids Res 2015, PMID: 26089390 (H. Sasanuma, Tokyo Metropolitan Institute of Medical Science, Japan)<br>TSCER2 EXO1-/-: Kratz et al. Mol Cell Biol 2021, PMID: 34228493 (H. Sasanuma, Tokyo Metropolitan Institute of Medical Science, Japan)<br>TSCER2 POLQ-/-: This paper (H. Sasanuma, Tokyo Metropolitan Institute of Medical Science, Japan)<br>TSCER2(TIR): Hoa et al. Genes Cells 2015, PMID: 26525166 (H. Sasanuma, Tokyo Metropolitan Institute of Medical Science, Japan)<br>TSCER2(TIR) CtIPCtIP-AID/CtIP-AID, Hoa et al. Genes Cells 2015, PMID: 26525166 (H. Sasanuma, Tokyo Metropolitan Institute of Medical Science, Japan)<br>TSCER2(TIR) BRCA1BRCA1-AID/BRCA1-AID: Sasanuma et al. Proc Natl Acad Sci U S A 2018, PMID: 30352856 (H. Sasanuma, Tokyo Metropolitan Institute of Medical Science, Japan)<br>TSCER2(TIR) BRCA2BRCA2-AID/BRCA2-AID/BRCA2-AID: This paper (H. Sasanuma, Tokyo Metropolitan Institute of Medical Science, Japan)<br>AP39P: JCRB, JCRB3068<br>FA18JTO: JCRB, JCRB0315<br>FA18JTO hTERT: JCRB, JCRB3007<br>AP66S hTERT, JCRB, JCRB3066
Authentication	Cell lines were authenticated by JCRB at the point of purchase.
Mycoplasma contamination	The cell lines utilized in this study tested negative for mycoplasma at the time of purchase. Any mycoplasma-positive samples were decontaminated prior to delivery to our laboratory. Mycoplasma contamination was not necessarily confirmed for each individual experiment.
Commonly misidentified lines<br>(See ICLAC register)	No cell lines featured in the database of commonly misidentified lines were utilized in this study.

Flow Cytometry

Plots

Confirm that:

☒ The axis labels state the marker and fluorochrome used (e.g. CD4-FITC).

☒ The axis scales are clearly visible. Include numbers along axes only for bottom left plot of group (a 'group' is an analysis of identical markers).

☒ All plots are contour plots with outliers or pseudocolor plots.

☒ A numerical value for number of cells or percentage (with statistics) is provided.

Methodology

Sample preparation	Cultured cell lines were collected from cell culture plates. No antibodies were used for flow cytometry in this study.
Instrument	BD FACS Aria III, FACS Fusion, or FACS Aria IIu (Becton DIckinson)
Software	FACS Diva (version 8.0.1; BD)
Cell population abundance	All collected cells from a well were sorted, and more than 20,000 cells were tested.
Gating strategy	FSC-SSC gating was used.

☒ Tick this box to confirm that a figure exemplifying the gating strategy is provided in the Supplementary Information.
