# Supplementary material for: Inducing multiple nicks promotes interhomolog homologous recombination to correct heterozygous mutations in somatic cells
Source: Nat Commun. 2023 Sep 15;14:5607. doi: 10.1038/s41467-023-41048-5 (PMC10504326; doi:10.1038/s41467-023-41048-5)
Supplement: Supplementary file 3 — Description of Additional Supplementary Files [file 41467_2023_41048_MOESM3_ESM.pdf]

Title: Supplementary Data 1:
Description: List of guide RNAs

Title: Supplementary Data 2:
Description: List of predicted off-targets (sgEx4_mt20s)

Title: Supplementary Data 3:
Description: List of predicted off-targets (sgS8)

Title: Supplementary Data 4:
Description: List of predicted off-targets (sgS14)

Title: Supplementary Data 5:
Description: List of predicted off-targets (sgS34)

Title: Supplementary Data 6:
Description: List of cell lines and culture media

Title: Supplementary Data 7:
Description: List of oligonucleotides

Title: Supplementary Data 8:
Description: List of antibodies

Title: Supplementary Data 9:
Description: List of amplicon-based NGS BAM files

Title: Supplementary Data 10:
Description: List of WGS data
